# Optimal Conservation Outcomes Require Both Restoration and Protection

**DOI:** 10.1371/journal.pbio.1002052

**Published:** 2015-01-27

**Authors:** Hugh P. Possingham, Michael Bode, Carissa J. Klein

**Affiliations:** 1 Australian Research Council Centre of Excellence for Environmental Decisions, School of Biological Sciences, University of Queensland, Brisbane, Australia; 2 Imperial College London, Department of Life Sciences, Silwood Park, Berkshire, England, United Kingdom; 3 Australian Research Council Centre of Excellence for Environmental Decisions, School of Botany, University of Melbourne, Melbourne, Victoria, Australia; University College London, UNITED KINGDOM

## Abstract

Conservation outcomes are principally achieved through the protection of intact habitat or the restoration of degraded habitat. Restoration is generally considered a lower priority action than protection because protection is thought to provide superior outcomes, at lower costs, without the time delay required for restoration. Yet while it is broadly accepted that protected intact habitat safeguards more biodiversity and generates greater ecosystem services per unit area than restored habitat, conservation lacks a theory that can coherently compare the relative outcomes of the two actions. We use a dynamic landscape model to integrate these two actions into a unified conservation theory of protection and restoration. Using nonlinear benefit functions, we show that both actions are crucial components of a conservation strategy that seeks to optimise either biodiversity conservation or ecosystem services provision. In contrast to conservation orthodoxy, in some circumstances, restoration should be strongly preferred to protection. The relative priority of protection and restoration depends on their costs and also on the different time lags that are inherent to both protection and restoration. We derive a simple and easy-to-interpret heuristic that integrates these factors into a single equation that applies equally to biodiversity conservation and ecosystem service objectives. We use two examples to illustrate the theory: bird conservation in tropical rainforests and coastal defence provided by mangrove forests.

## Abstract 2

In conservation, prevention is not always better than cure, either for protecting biodiversity or ecosystem services. It can be better to start habitat restoration before all available intact habitat has been protected.

## Author Summary

Most species go extinct because humans have cleared their habitat. Habitat loss can also cause people to lose some of the services provided by ecosystems, such as the removal of carbon dioxide from the atmosphere or the protection of coastal communities from storm damage. There are two broad strategies for stopping and reversing habitat loss: we can either protect habitat that is currently intact, or we can restore habitat that has already been cleared. Superficially, we might imagine that, as with human health, “prevention is better than cure,” and that therefore habitat protection should be given priority over habitat restoration. However, there is currently no scientific theory to justify this belief. Here, we used an ecosystem model and dynamic optimization tools from mathematics to show that habitat restoration (such as tree planting) can, surprisingly, be more cost-effective than habitat protection (such as designating a national park) for two case studies. We discovered that the best decision depends on the relative costs of the two actions, the rate at which habitat is being lost, and the time lag between restored habitat being as useful as intact habitat for securing species and ecosystem services.

## Introduction

Habitat conservation is central to biodiversity conservation. Habitat can be conserved by either protecting it if it remains intact or by restoring it once it has been degraded. Conservation organisations often pursue both restoration and protection simultaneously, and management guidelines advocate the use of both actions [1,2]. However, the orthodox position is that managers should “protect first, restore second” where possible, and the priority of protection has been argued in the scientific literature [3–7] and management guidelines internationally [8–10], for both biodiversity conservation and ecosystem service provision. This prioritisation of investment in protection over restoration is justified with reference to the relative costs, expected benefits, and timescales of the two actions [3–12]. While restoration can improve a site’s ecological condition, restored habitat will often take decades to regain the majority of its biodiversity and ecosystem attributes [3,13].

Despite this prevailing wisdom, recent experiments have revealed that the disparity between restoration and protection is smaller than expected, and strongly context dependent. The majority of many ecosystem features, particularly certain ecosystem services, can be provided by restored habitat [3], sometimes within a surprisingly short timeframe [14,15]. Moreover, ongoing technological advances [16] mean that the cost of this restoration can be low enough to generate a net social benefit [11,17]. At the same time, conservation theory has highlighted disadvantages to habitat protection that parallel problems identified for restoration. Protected areas also suffer from poor implementation and management and do not guarantee the conservation of intact habitat, species assemblages, or ecosystem services [18,19]. Dynamic landscape models further illustrate how the benefits of protection are also subject to time delays, since protection does not create new habitat, but only reduces the likelihood of future habitat loss [20]. Moreover, in highly degraded landscapes that leave many species with non-viable populations (i.e., extinction debts [21]), habitat protection will only have a secondary effect on biodiversity loss rates. Restoration is the only in situ conservation intervention that can actively reduce an extinction debt [22,23].

The relative priority of protection and restoration can only be coherently assessed by a conservation resource allocation theory that incorporates both actions and that quantifies both their costs and benefits in a comparable manner. In particular, this theory must be temporally explicit, since both the benefits of the two actions accrue at different rates. In this paper, we incorporate restoration and protection into a shared dynamic landscape model [20] that explicitly includes restoration rates, dynamic species loss (i.e., extinction debts), and ecosystem service provision (see Materials and Methods). To contrast the performance of habitat protection and restoration, we apply this unified theory to two divergent examples: ecosystem service provision in the Coral Triangle and biodiversity conservation in the Atlantic forests.

## Results

### Example 1: Coastal Defence in the Coral Triangle

Intact mangrove ecosystems help defend coastal communities from storm surges and floods [24,25], events that are increasing in frequency and intensity as the climate changes [26]. In Southeast Asia, the impact of frequent extreme weather events on coastal communities is pronounced due to vulnerable infrastructure and high rates of mangrove deforestation [27]. We focused on the northern tip of Borneo (Sabah, Malaysia), where over 38,000 people live on the coast. Approximately 78% of mangrove forests remain from an original 535 km^2^, after more than 30 years of losing 0.5%–0.8% annually to other uses (e.g., urban development and aquaculture) [27]. The Sabah Forestry Department is charged with both the protection and restoration of mangrove forests, two actions that gained popularity across Southeast Asia after the 2004 Indian Ocean tsunami, but they lack a decision framework for deciding which action is best to implement [28].

Barbier et al. [25] calculated that coastal defence benefits accrue as a nonlinear function of the area of surrounding intact mangrove habitat:

E(t)=1−exp(−k(P(t)+F(t))) Eq (1)

where *P*(*t*) is the area of protected mangrove forest, *F*(*t*) is the area of unprotected but intact mangrove forest, and *k* = 2.1x10^–3^ (S1 Text). Eq (1) measures ecosystem service provision by the proportional reduction in damage caused by a lower wave height in areas sheltered by mangrove forests. In this example, over the course of a 30-year project starting in 2006, managers must choose to share an annual conservation budget equivalent to US$15 million between the protection of intact mangrove forests, the restoration (and subsequent protection) of degraded mangrove habitat, or a combination of both actions. Their aim is to maximize the total coastal defence provided to communities by mangrove forests over the project lifetime:

$$\max_{u(t)}\int_{t=0}^{T}e^{-\mathrm{rt}}[1-e^{-k(P(t)+F(t))}]\mathrm{dt}$$ Eq (2)

where *r* is the economic discount rate. We assume that restored mangrove forests do not contribute to coastal defence until restoration is complete (see S1 Text for parameterisation details).

**Fig 1:**
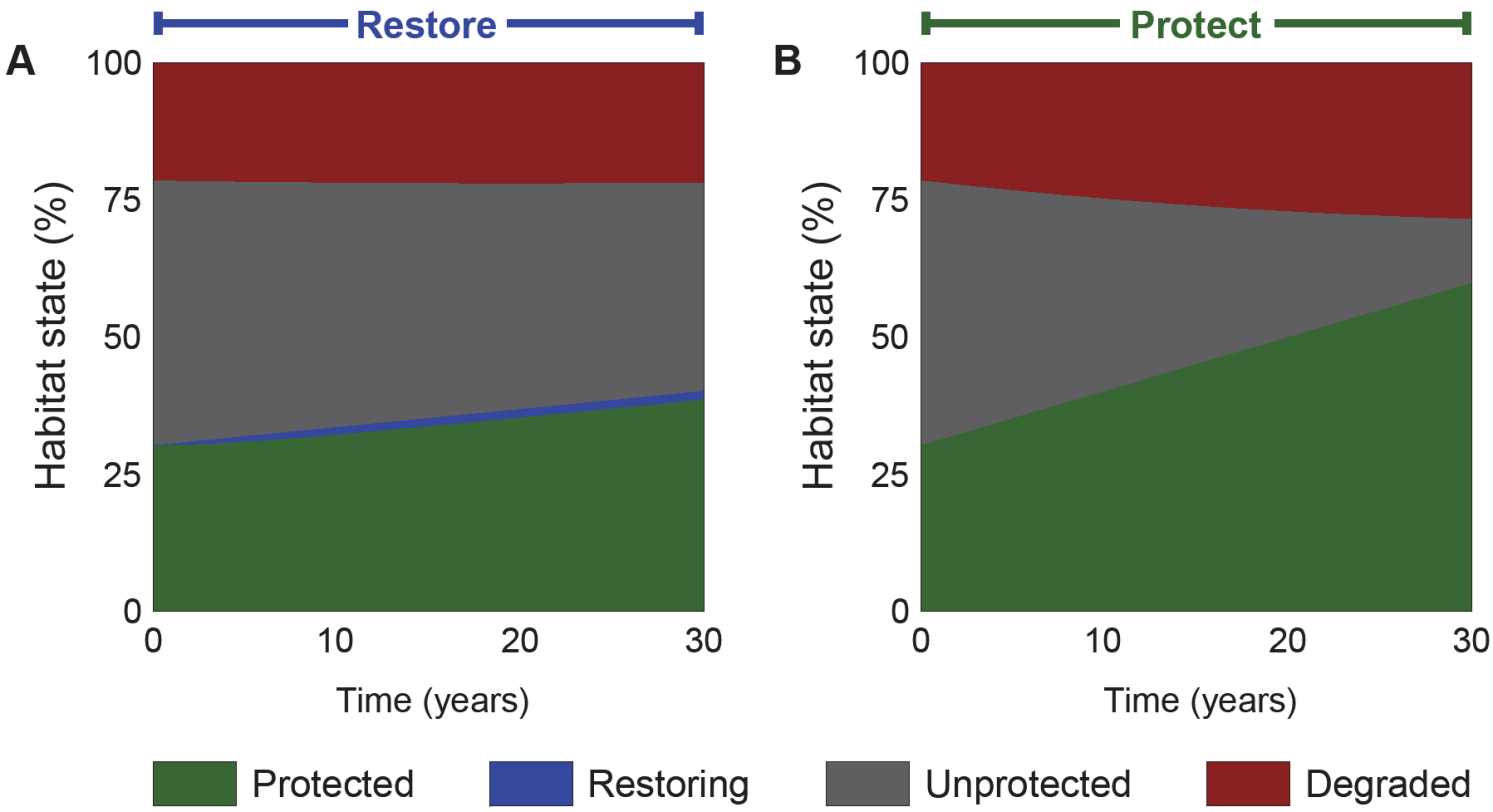
Allocation schedules for mangrove ecosystem service provision. Proportion of landscape in each land state over a 30-year mangrove conservation project in Sabah. Results show (A) the optimal allocation decision to only restore, and (B) the standard approach of pursuing protection while intact habitat remains unprotected. Prioritising restoration results in fewer protected areas (green) and a greater amount of unprotected intact habitat (grey), but has greater success limiting the amount of degraded land (red), therefore maximising the provision of ecosystem services (see Fig. 2A). The data used in this figure is given in S1 Data, and the Matlab code that generated it can be found in S2 Text.

**Fig 2:**
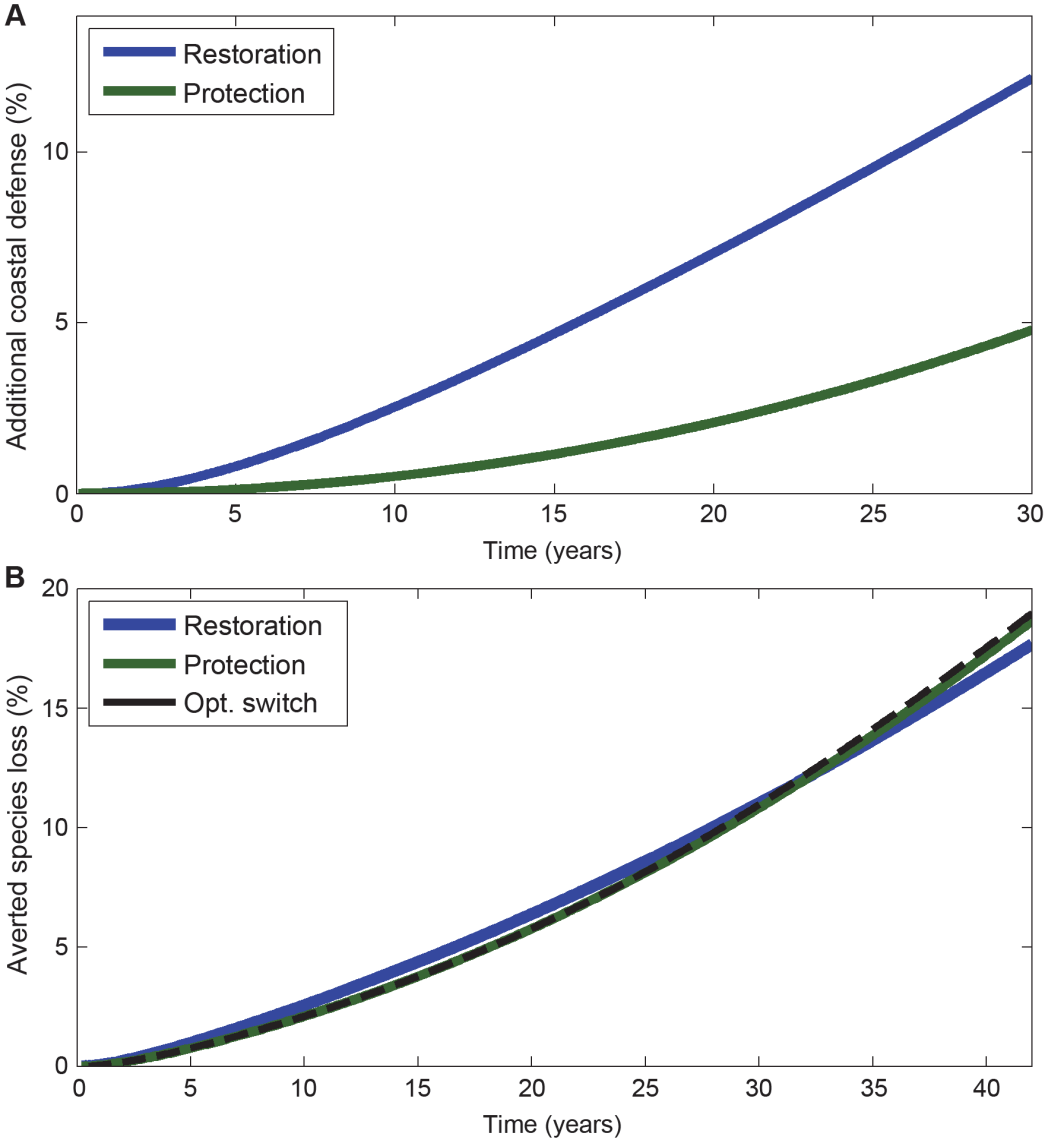
Performance of alternative allocation schedules. The conservation outcomes delivered by different allocation schedules, both measured relative to a null model of no management investment. (A) Annual provision of additional ecosystem services from mangrove conservation in Sabah under the optimal allocation (restoration-only, blue line), and the standard, protection-only approach (green line). Performance is measured relative to the amount of coastal defence that would be expected in the absence of management intervention. (B) Extinctions averted by the optimal allocation schedule, which switches from protection to restoration after 20 years (dashed black line). Results are also shown for the standard, protection-only approach (green line), and a restoration-only approach (blue line). The number of observed extinctions is reported in comparison to the number of extinctions we would expect in the absence of management intervention. The data used in this figure is given in S2 Data, and the Matlab code that generated it can be found in S2 Text and S3 Text.

To maximise coastal defence, the model predicts that northern Sabah managers should prioritise the restoration of cleared or degraded mangrove habitat over the protection of intact forests (Fig. 1A and Fig. 2A). The optimal allocation schedule suggests that restoration should be an absolute priority (i.e., all available funds should be directed towards restoration), even though the optimisation method allows managers to allocate part of their funds to both actions (e.g., 95% to restoration and 5% to protection). Restoration results in a smaller amount of protected forest than protection (Fig. 1B), both because restoration is significantly more expensive, and because restored habitat only becomes intact and protected after a substantial time lag. Nevertheless, restoration provides the local community with more coastal defence because it results in less degraded land and more intact forest (protected and unprotected; Fig. 2A). The optimal allocation therefore assigns a higher priority to restoration.

### Example 2: Biodiversity Conservation in Paraguay’s Atlantic Forests

Paraguay contains some of the last remnants of South America’s high-latitude tropical rainforests, which remained predominantly intact into the 1970s [29]. Given the extraordinary endemic bird species richness (148 species) and high levels of habitat loss (>90%) in this ecoregion, the small number of extinctions to date indicates the presence of a substantial extinction debt [30]. Managers want to minimise the number of bird extinctions by either restoring and then protecting cleared or degraded habitat, by protecting the last remaining stands of intact rainforest, or by a combination of the two [31]. The species–area relationship relates the equilibrium species richness to the area of intact habitat, regardless of its protection status:

$$S^{*}=\alpha {\left(P+F\right)}^{z}$$ Eq (3)

where α represents regional species richness and *z* is a constant. We make the conservative assumption that restored habitat does not mitigate the extinction debt until restoration is complete, and we estimate the recovery rate using long-term surveys of species richness following forest clearance (although the complete recovery of population abundances will take longer than the return of species; S1 Text). Species extinctions often lag significantly behind habitat loss, but debt “relaxation” can be averted if degraded habitat is quickly restored. The rate of species extinction is modelled as proportional to the size of the species debt, calculated as the difference between extant species richness and the number of species that would be supported by the current habitat distribution at equilibrium [32]:

$$\frac{\mathrm{dS}(t)}{\mathrm{dt}}=\theta [S(t)-S^{*}]=\theta [S(t)-\alpha {\left(P(t)+F(t)\right)}^{z}],$$ Eq (4)

where *θ* is the rate of extinction debt relaxation. The managers’ objective is to minimize the total number of extinctions during a *T* year conservation project:

$$\min_{u(t)}\int_{t=0}^{T}[S(t)-\alpha {\left(P(t)+F(t)\right)}^{z}]\mathrm{dt}$$ Eq (5)

**Fig 3:**
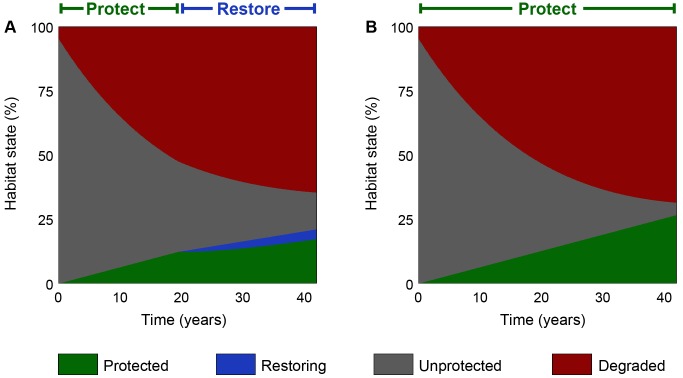
Allocation schedules for rainforest biodiversity conservation. Proportion of landscape in each land state through time over 43 years of rainforest conservation in Paraguay’s Atlantic Forest (1970–2013). Results show (A) the optimal allocation schedule of protection for 20 years, followed by 23 years of restoration, and (B) the standard approach of protection while intact habitat remains unprotected. An early switch from protection to restoration results in fewer protected areas (green) and more unprotected habitat (grey), but less degraded land (red). The result is fewer species extinctions (see Fig. 2B). The data used in this figure is given in S3 Data, and the Matlab code that generated it can be found in S3 Text.

We calculated a retrospective optimal management strategy for protection and restoration between 1970–2013 (Fig. 3; see S1 Text for parameterisation details), assuming an ongoing annual budget equivalent to US$100 million (2014). The results show that, in the Atlantic Forests, restoration and protection would have achieved broadly comparable outcomes (Fig. 2B). However, to optimally reduce species extinctions over the time period, managers should have pursued habitat protection for the first 20 years and then switched their efforts entirely towards restoration (Fig. 3A). It is not optimal to fund both actions simultaneously, at any point in time.

Rates of habitat degradation in Paraguay’s Atlantic Forests were so rapid that neither action would have had a large impact on the size of the extinction debt. Nevertheless, an initial focus on protection would have quickly reduced the amount of habitat that was unprotected, and that therefore could be degraded. Once this was achieved, a switch to restoration would have allowed managers to address the extinction debt directly, by converting degraded land back into intact habitat.

### Rule of Thumb

We identified the best allocation schedules using optimal control theory, but the relative benefits of protection and reservation can often be better understood and implemented using myopic heuristics [20]. Both biodiversity and ecosystem service objectives are advanced by an increase in the total amount of intact habitat (protected and unprotected). The act of protection increases intact habitat indirectly by reducing the amount of unprotected habitat available for degradation, while restoration converts degraded land to protected intact habitat, after a time delay. A linearisation of the state equations (Eq 7; Materials and Methods) indicates that protection should be a higher conservation priority if half the ratio of the restoration and habitat loss rates is less than the ratio of their costs (see S1 Text for derivation):

$$\frac{g}{2\delta }<\frac{c_{R}}{c_{P}}$$ Eq (6)

Roughly speaking, restoration is favoured if its relatively higher costs (*c*_*R*_/*c*_*P*_) are outweighed by its more rapid reduction of degraded land relative to protection (*g*/2*δ*). In agreement with our optimal control solutions (Fig. 1 and Fig. 3), Eq (6) predicts that restoration should be strongly favoured over protection in mangrove forests (*g*/2*δ* = 13.1; *c*_*R*_/*c*_*P*_ = 3.0). In contrast, protection should be considered a slightly higher priority than restoration in the Atlantic Forests (*g*/2*δ* = 1.1; *c*_*R*_/*c*_*P*_ = 1.6; see S1 Text for parameter values).

This simple rule of thumb holds true for both ecosystem service provision and biodiversity conservation objectives. The right-hand side of this condition is familiar—relative costs of action are consistently identified as important elements of prioritisation [33–35]. However, the left-hand side highlights another important factor in conservation planning: the timescales over which interventions yield benefits. An interpretation of this temporal ratio is that restoration will become a higher priority when habitat quickly regains its pristine qualities; protection should be preferred when the restoration rate is slow or when the rate of land degradation is rapid. This rule can be readily modified to include the probability that restoration and protection will be unsuccessful; the results of this modification indicate that including failure rates are equivalent to increases in the costs of the respective action (S1 Text). For example, if the probability of restoration being successful was only 50%, then that is equivalent to a doubling of the cost of restoration.

## Discussion

Constrained by limited budgets, conservation organisations and governments must always choose between restoration and protection. Our framework provides a coherent framework that can help resolve longstanding uncertainties about the relative priority of these two fundamental actions. The results and implications are much more complex than simply “protect first, restore second.” In both of our examples, the long-term objectives will be best achieved if priority is given to restoration at some point in the project, despite its higher cost, and despite a substantial delay before restored habitat can contribute to project objectives.

As well as demonstrating the benefits of restoration, our results indicate that the optimal solution is always to spend all available resources on either restoration or protection, never both. While the option of splitting the budget between the two actions was available to our optimisation method, it was never optimal to fund both actions simultaneously. This type of either-or solution—known as bang-bang control—is often sensitive to model assumptions about homogeneity, uncertainty, and linearity. Our model makes all of these assumptions. The model in Eq (7) considers only the overall landscape scale and contains no fine-resolution ecological or economic variation, with all land incurring the same restoration or protection costs. In reality, conservation costs (and benefits) vary dramatically between locations and projects [36]. We assume that the rates of land loss and restoration are known and deterministic, but land degradation is a stochastic process, and depends on highly uncertain factors, as do both restoration success and protected area performance. Finally, the management control terms in Eq (7) are linear in the control variable *u*(*t*) (e.g., *uB*/*c*_*P*_), while in reality, expenditure generally achieves diminishing marginal returns [37]. Relaxing any of these assumptions will tend to smooth the abrupt switch between restoration and protection—that is, will create a more continuous transition period during which both actions are funded simultaneously.

Our objective functions (Eqs 2 and 5) aim to maximise unidimensional descriptions of biodiversity conservation (the number of bird species) and ecosystem service provision (the amount of coastal protection). For individual ecosystem services like coastal protection or carbon sequestration, this is a reasonable formulation of conservation objectives. However, many conservation agencies carry out projects that aim to deliver multiple benefits simultaneously, and almost all conservation actions will inevitably provide benefits to more than one ecosystem service or measure of biodiversity. It is possible, for example, to imagine a conservation project that pursues both our stated objectives—coastal protection and species conservation—simultaneously. While it is possible to aggregate the provision of multiple benefits into unidimensional quantities by calculating the monetary value of different ecosystem services [25] or by assigning relative weightings to different species [38], this is not always appropriate or desirable. The process of aggregation will also be further complicated by the divergent values of multiple stakeholders [39]. In such situations, optimisation may not be as useful as an exploration of how restoration and protection affect trade-offs between objectives and conflict between stakeholders.

Our broad, qualitative conclusion—that protection should not always be prioritised over restoration—is robust to the parameterisation of our two examples and to the structure of the restoration process (S1 Text). However, both restoration and protection are more complicated and nuanced than any of the abstracted models we apply here. Recent meta-analyses of terrestrial and aquatic restoration projects show that even successful restoration projects are unable to recover reference-level biodiversity and ecosystem services (they achieved an average of 80%–86% of reference sites, although technological improvements continue to improve these outcomes [3,16]). Moreover, these benefits accrue to different ecosystem features at varying timescales, and may take decades to be realised, particularly for the restoration of biodiversity [40,41]. Alternate versions of the landscape model can incorporate incomplete restoration (one alternative formulation is given in the S1 Text), but at the cost of increased complexity and greater information requirements. Protection is also rarely perfectly effective, both because effectively managed protected areas cannot halt all degrading activities [18] and because many protected areas are poorly managed [42]. Furthermore, decisions to restore or protect are influenced by a variety of important factors not considered here, including the feasibility of actions in a given place, which is influenced by operational (e.g., technical success), legal (e.g., land tenure), political (e.g., political will), and social constraints (e.g., the willingness of landowners). In particular, our nonspatial model omits the constraints placed on managers by spatial conservation objectives. For example, if managers want to connect particular areas or reduce fragmentation in a landscape, restoration will be the only suitable action. Despite these omissions, we believe that simple, general theory can still provide useful insights into a problem.

By providing a unified, dynamic framework within which to compare their long-term outcomes, our theory provides evidence and rationale for pursuing restoration alongside protection—even in preference to protection—under the right circumstances. An explicit theoretical framework also helps to highlight relationships that determine the priority of the two actions: the relative costs of restoration and protection, and the rate at which restored habitat approaches the benefits of intact habitat relative to the habitat conversion rate. Our two examples illustrate this general theory and demonstrate the unexpected potential for restoration to parallel or precede protection. However, the approach and simple rule of thumb should be seen as informative, not prescriptive.

## Materials and Methods

Our framework is not intended to inform precisely where protection and restoration should occur at a fine scale within a landscape, but to offer insights into the allocation of a limited conservation budget at a meso-scale (e.g., bioregional or catchment conservation initiatives). The model is nonspatial, assuming that the conservation state of the landscape can be described by the aggregate proportions of the land in different states (e.g., the proportion protected, degraded, etc.). Actual decisions about precisely where to act are inherently spatial and should be informed using spatial zoning tools that optimize for multiple conservation actions [43].

## Dynamic Conservation Landscape Model

The decision about whether to restore or protect is underpinned by a basic dynamic landscape model. Each small area of land at time *t* is classified as being in one of four states: intact and unprotected, *F*(*t*); intact and protected, *P*(*t*); degraded or cleared, *C*(*t*); or undergoing restoration, *R*(*t*). We describe the total amount of land in each state as a proportion of the landscape, and the model ensures that *F*(*t*) + *P*(*t*) + *C*(*t*) + *R*(*t*) = 1 at all times.

In our model, transitions of land between the four states are driven by two actions and two processes. The two actions, protection and restoration, are entirely determined by the managers. At each point in time across a *T*-year project, managers can allocate a varying proportion (0≤*u*(*t*)≤1) of a fixed annual budget (*B*) to protection, and the remainder 1—*u*(*t*) to restoration. We note that this choice of *u*(*t*) allows managers to simultaneously fund both actions—that is, to allocate a proportion of their resources to protection, and the remainder to restoration (e.g., if *u*(*t*) = 0.1, managers spend 10% of their budget on protection and 90% on restoration). Managers cannot directly alter the two processes. The first process is land degradation, in which we assume that unprotected land is being degraded at proportional rate *δ* [20,44,45]. That is, landscapes with large amounts of intact, unprotected habitat will experience large absolute rates of habitat loss. Managers can therefore reduce habitat loss by decreasing the amount of unprotected habitat through protection, but they cannot directly affect the loss rate *δ*. We note that alternative models of land degradation (e.g., constant rates) could also be used. The second process is restoration. We assume that restoration actions, once undertaken, do not create intact habitat immediately. Once managers spend resources by purchasing degraded land and undertaking restoration actions, the land undergoing restoration, *R*(*t*), only regains its intact qualities at a proportional rate *g*. We note that this is a continuous model of restoration, rather than a time-lag model, but show in the S1 Text that this simplification does not qualitatively alter our conclusions.

By combining these actions and processes, the rate of change of each land state becomes:

$$\begin{array}{l} \frac{dF\left(t\right)}{dt}=-\delta F-\frac{u\left(t\right)B}{c_{P}}\\ \frac{dP\left(t\right)}{dt}=\frac{u\left(t\right)B}{c_{P}}+gR\\ \frac{dC\left(t\right)}{dt}=\delta F-\frac{\left(1-u\left(t\right)\right)B}{c_{R}}\\ \frac{dR\left(t\right)}{dt}=\frac{\left(1-u\left(t\right)\right)B}{c_{R}}-gR \end{array}$$ Eq (7)

**Fig 4:**
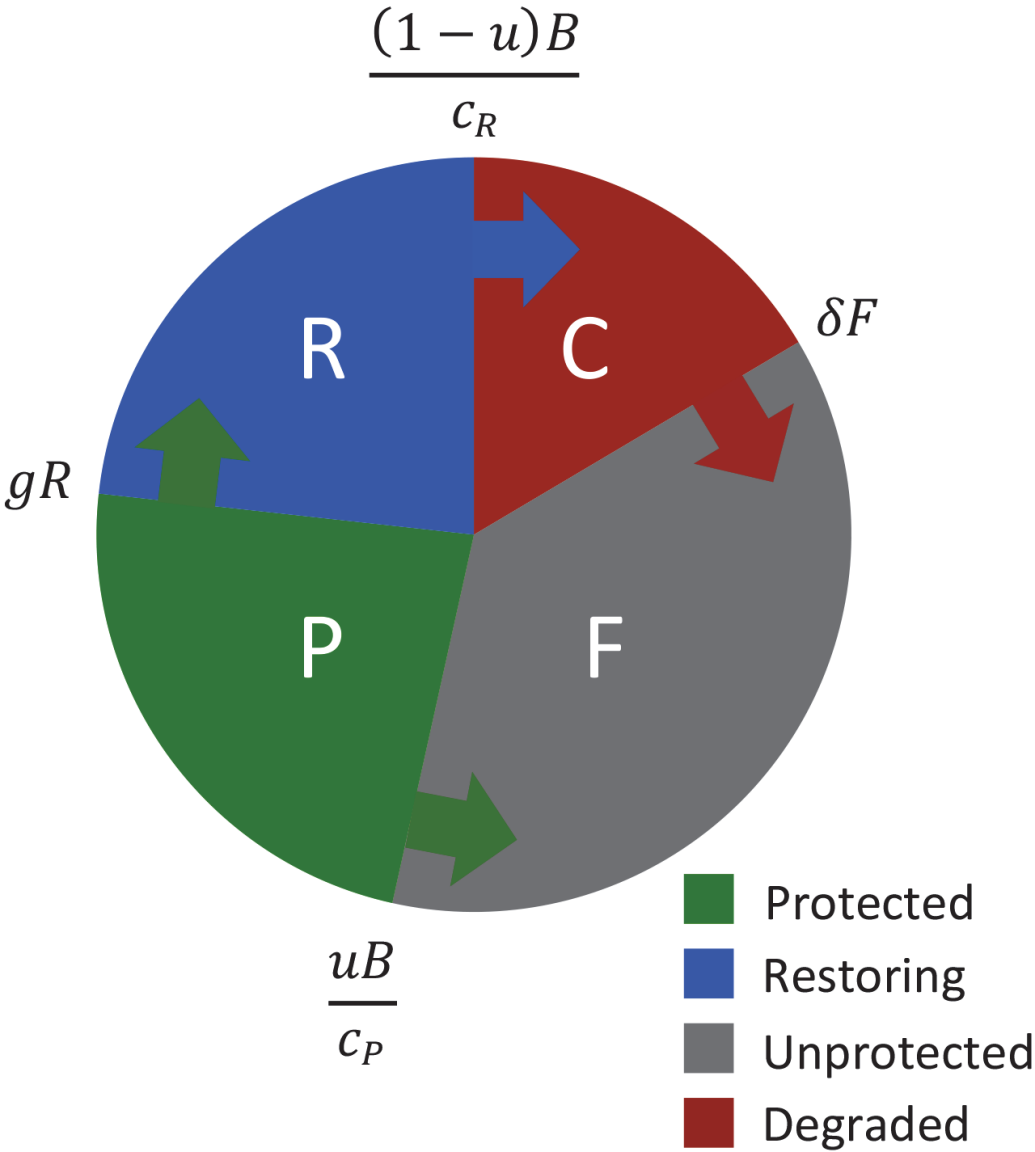
Dynamics of the unified restoration and protection model. Schematic representation of the landscape dynamics described by Eq (7). Arrows show the direction of state changes between the four land states, with the mathematical terms indicating the magnitude of the flux. For example, the blue arrow extending from restoring land (R) to degraded land (C) indicates that the process of restoration moves land from the “degraded” state to the “restoring” state.

The variables *c*_*P*_ and *c*_*R*_ denote the costs of protection and restoration respectively. Because habitat must be purchased before it is restored, *c*_*R*_ is generally larger than *c*_*P*_. However, if land has been abandoned, or if the primary value of land comes from the intact habitat itself (e.g., timber), then purchasing degraded land for restoration may be cheaper than purchasing intact habitat for protection. The protection or restoration of a land parcel both require ongoing management, and we therefore assume that these two cost parameters measure the endowed cost of undertaking both actions until the end of the project. That is, *c*_*P*_ is equal to the initial purchase price of the land, plus the time discounted cost of all future actions required to effectively manage the protected area. Likewise, *c*_*R*_ is the sum of the land purchase price, the time discounted cost of the initial intensive restoration actions, and then the time discounted cost of ensuring that the restoration and subsequent protection are effective. The entirety of these endowed costs must be paid in the year that a given parcel of land is restored or protected. The dynamics described by Eq (7) are illustrated in Fig. 4.

Identifying the optimal management schedule is equivalent to determining the specific control function *u*(*t*) that maximises the objective function. There are clearly a very large number of candidate control functions, but fortunately the optimal function can be identified by applying Pontryagin’s maximum principle [46,47] to the system dynamics in Eq (3), and each problem’s objective function (Eq 1–2). The details of this analysis are shown in the S1 Text. We note that it is not essential to understand the specifics of this optimisation method since alternative methods could equally be used to solve for the optimal solution, notably stochastic dynamic programming [20].

## Supporting Information

S1 Data Spreadsheet containing the data illustrated in Fig. 1. (CSV)

S2 Data Spreadsheet containing the data illustrated in Fig. 2. (CSV)

S3 Data Spreadsheet containing the data illustrated in Fig. 3. (CSV)

S4 Data Spreadsheet containing the data illustrated in S2 Fig. (CSV)

S5 Data Spreadsheet containing the data illustrated in S3 Fig. (CSV)

S6 Data Spreadsheet containing the data illustrated in S4 Fig. (CSV)

S7 Data Spreadsheet containing the data illustrated in S5 Fig. (CSV)

S8 Data Spreadsheet containing the data illustrated in S6 Fig. (CSV)

S9 Data Spreadsheet containing the data illustrated in S7 Fig. (CSV)

S10 Data Spreadsheet containing the data illustrated in S8 Fig. (CSV)

S11 Data Spreadsheet containing the data illustrated in S9 Fig. (CSV)

S1 Fig Flow chart of the dynamic landscape model. Arrows show the direction of state changes between the four land states. Flux rates correspond to processes contained in Eq. (S1) of S1 Text.
(TIF)

S2 Fig Percent intact forest cover for Paraguay’s Atlantic Forests in three years sampled by Huang et al. [29] is shown using black circles. Best-fit constant proportional loss rate model (i.e., exponential decline in unprotected intact habitat) is shown with the grey line. The underlying data in this figure is given in S4 Data.
(TIF)

S3 Fig Number of species found in subtropical moist and wet rainforest in Carite (grey) and Luquillo (black) over a period of 81 years, during which the habitat is undergoing passive restoration. Best-fit asymptotic exponential recovery trajectories to each dataset are shown with correspondingly coloured lines. The underlying data in this figure was sourced from Aide et al. [31] and is given in S5 Data.
(TIF)

S4 Fig Habitat state distribution resulting from the optimal resource allocation schedules for mangrove conservation in Sabah. Results are shown for three different values of *g*, the rate of restoration. These are (A) the nominal estimate, g* = 0.21; (B) 75% of the nominal value, *g* = 0.16; and (C) 50% of the nominal value, *g* = 0.10. Optimal schedules are very similar, and in all cases give priority to restoration. Faster restoration rates simply mean that less habitat remains in the restoring state at any given time. The data used in this figure is given in S6 Data, and the Matlab code that generated it can be found in S2 Text.
(TIF)

S5 Fig Habitat state distribution resulting from the optimal resource allocation schedules for biodiversity conservation in Paraguay’s Atlantic Forests. Results are shown for three different values of *g*, the rate of restoration. These are (A) the nominal estimate, g* = 0.089; (B) 80% of the nominal value, *g* = 0.071; and (C) 120% of the nominal value, *g* = 0.107. The optimal schedules are qualitatively similar, beginning with protection, then switching resources to restoration after years have elapsed. However, for lower values of *g*, managers should spend more time protecting intact habitat before they shift across to restoring degraded habitat. The data used in this figure is given in S7 Data, and the Matlab code that generated it can be found in S3 Text.
(TIF)

S6 Fig Habitat state distribution resulting from the optimal allocation schedule for coastal protection by Sabah mangrove forests, when restoration incurs a fixed time delay. Resources are invested in restoration for approximately 13 years, after which time they are shifted across to protection for the remainder of the project duration. As with the continuous-restoration model, the schedule initially prioritises restoration. However, allocations differ in the latter years of the project, where optimisation of the fixed-delay model begins to protect land. This difference is partly due to the fact that any restoration in the last 14.5 years (the time lag of restoration) will produce no benefits since it will not be complete before the end of the project timeline. The data used in this figure is given in S8 Data, and the Matlab code that generated it can be found in S2 Text.
(TIF)

S7 Fig Habitat state distribution resulting from the optimal allocation schedule for biodiversity conservation in Paraguay’s Atlantic Forests, when restoration incurs a fixed time delay. Resources are initially invested in protection for 15 years, before switching to restoration for another period of approximately 15 years. Resources are finally allocated back to protection for the final 12 years of the project. This spending pattern begins with the same sequence of allocations as the continuous-restoration model (i.e., protect-then-restore), before changing in the final years to prioritise protection. This difference reflects the fact that restoration undertaken in the final years of the project will be incomplete (and therefore of no value) when the project ends. The data used in this figure is given in S9 Data, and the Matlab code that generated it can be found in S3 Text.
(TIF)

S8 Fig Habitat state distribution resulting from the optimal allocation schedules when costs are estimated differently for the Sabah mangrove forests example. (A) When managers preferentially restore and protect less expensive land (i.e., mangrove habitat that is not suitable for aquaculture), and protection costs are therefore reduced by 25% from its nominal value. This also reduces the cost of restoration, since it also requires the purchase of land. (B) When managers preferentially restore abandoned land, there is therefore no opportunity cost for restoration (only the cost of the restoration action). This reduces the cost of restoration but leaves the cost of protection unchanged. Neither alternate assumption changes the qualitative conclusions of our analysis. The data used in this figure is given in S10 Data, and the Matlab code that generated it can be found in S2 Text.
(TIF)

S9 Fig Percentage reduction in the height of storm-surge waves caused by a *Kandelia candel* mangrove stand of a given width *w*. The data in this figure was sourced from the supplementary information in Barbier et al. [25], and is given in S11 Data.
(TIF)

S1 Text Supplementary methods and references. (DOCX)

S2 Text Commented MATLAB code used to generate Fig. 1, Fig. 2, S4 Fig., S6 Fig., and S8 Fig. (DOCX)

S3 Text Commented MATLAB code used to generate Fig. 2, Fig. 3, S5 Fig., and S7 Fig. (DOCX)
