# Supplementary figures and images for: Optimal Conservation Outcomes Require Both Restoration and Protection
Source: PLoS Biol. 2015 Jan 27;13(1):e1002052. doi: 10.1371/journal.pbio.1002052 (PMC4308106; doi:10.1371/journal.pbio.1002052)

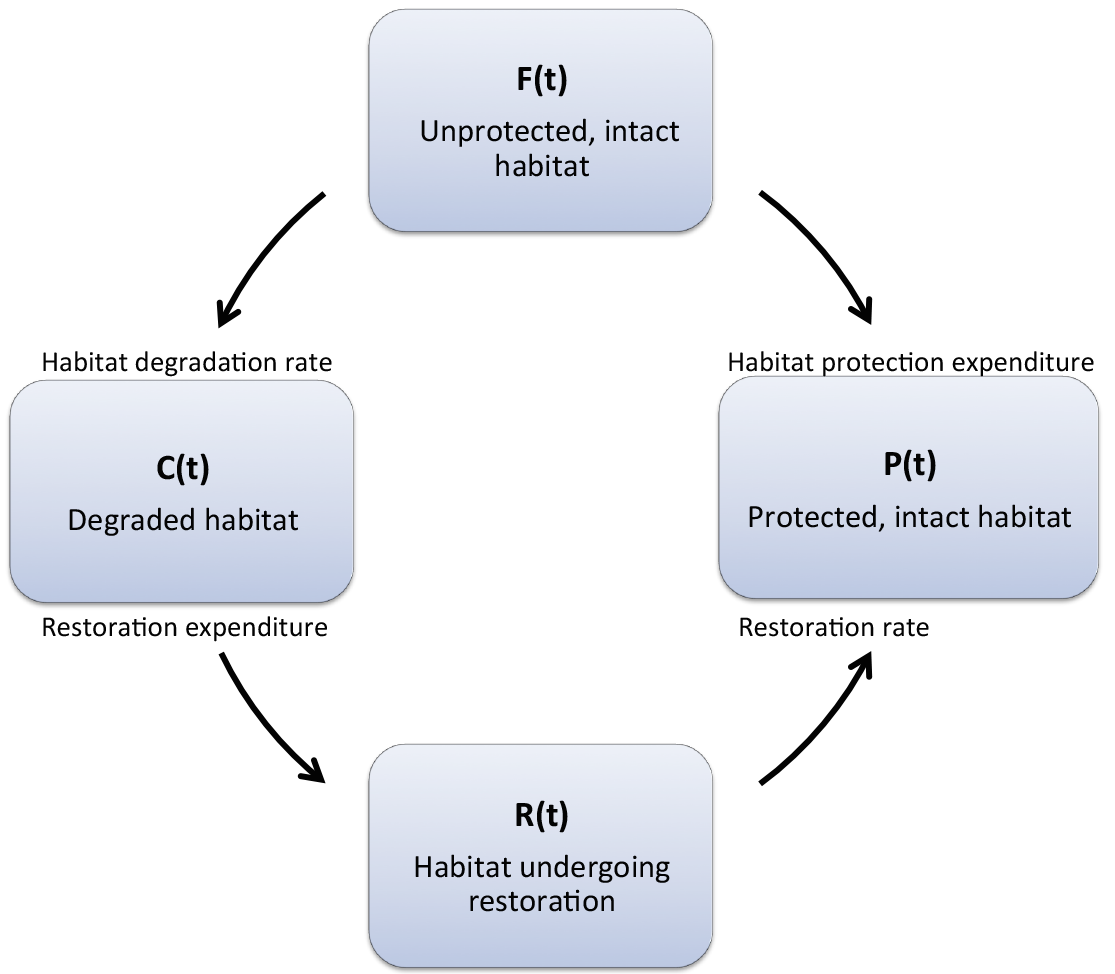

Supplement: S1 Fig — Arrows show the direction of state changes between the four land states. Flux rates correspond to processes contained in Eq. (S1) of S1 Text. (TIF) [file pbio.1002052.s012.tif]

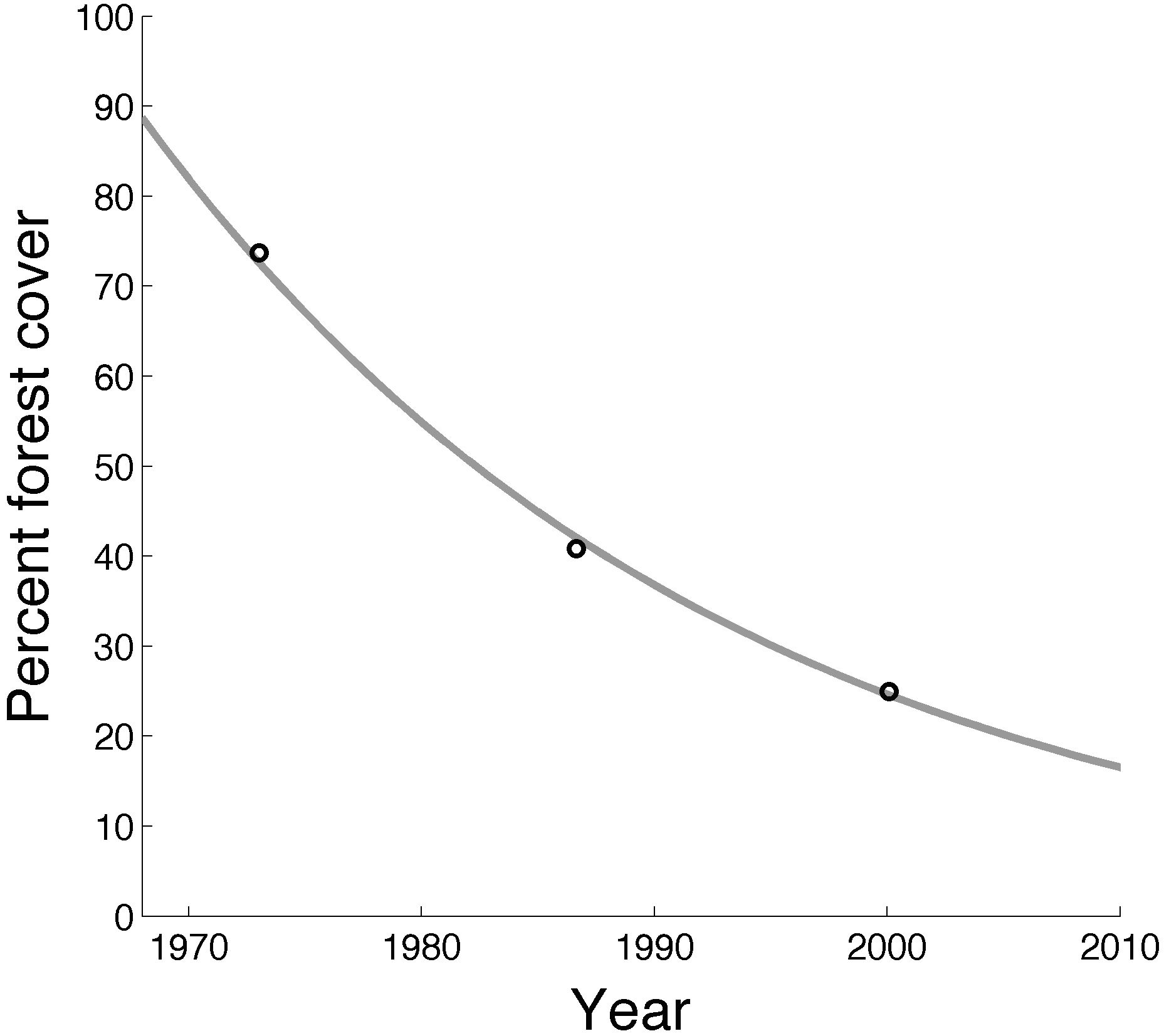

Supplement: S2 Fig — Best-fit constant proportional loss rate model (i.e., exponential decline in unprotected intact habitat) is shown with the grey line. The underlying data in this figure is given in S4 Data. (TIF) [file pbio.1002052.s013.tif]

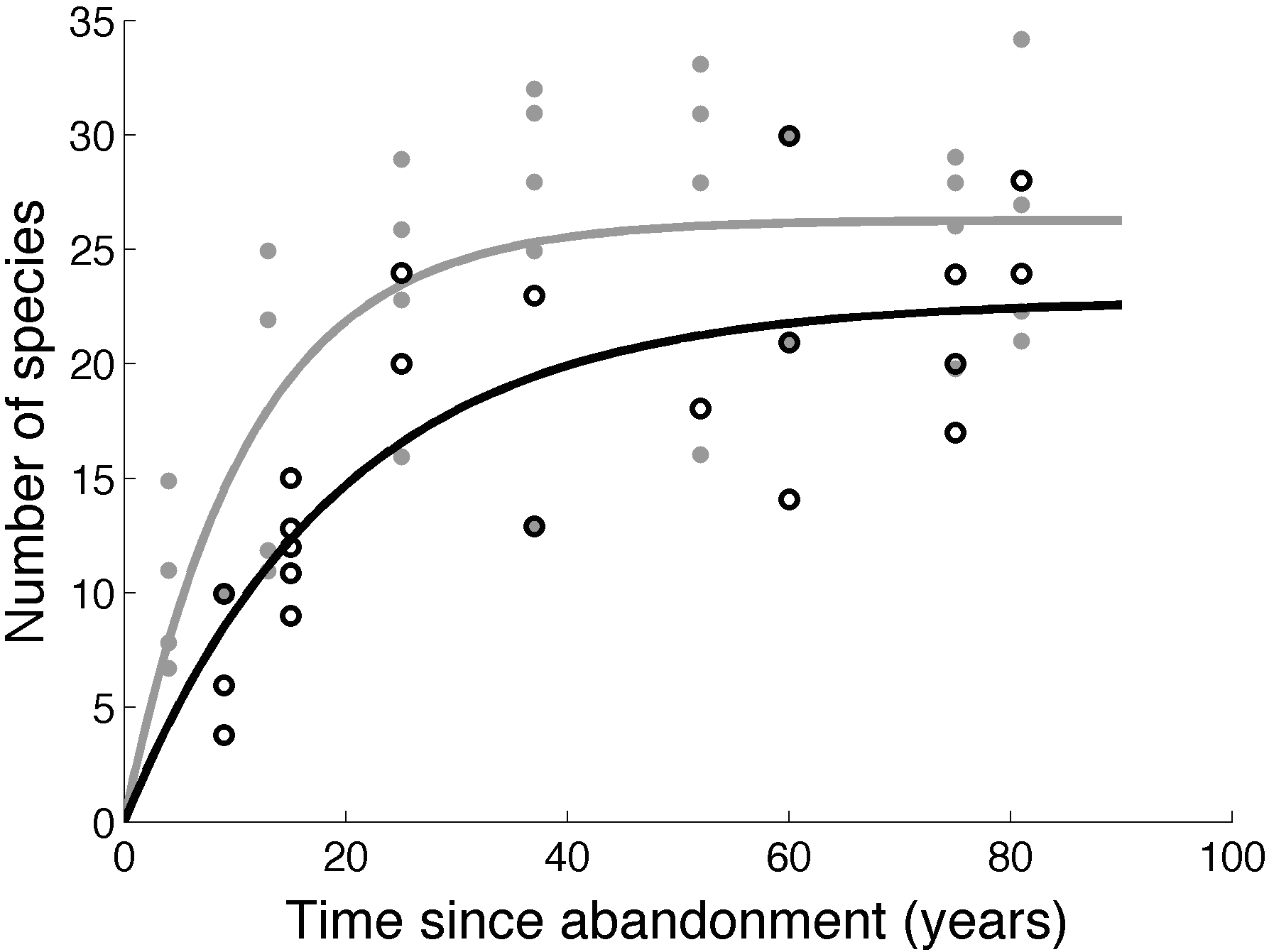

Supplement: S3 Fig — Best-fit asymptotic exponential recovery trajectories to each dataset are shown with correspondingly coloured lines. The underlying data in this figure was sourced from Aide et al. [31] and is given in S5 Data. (TIF) [file pbio.1002052.s014.tif]

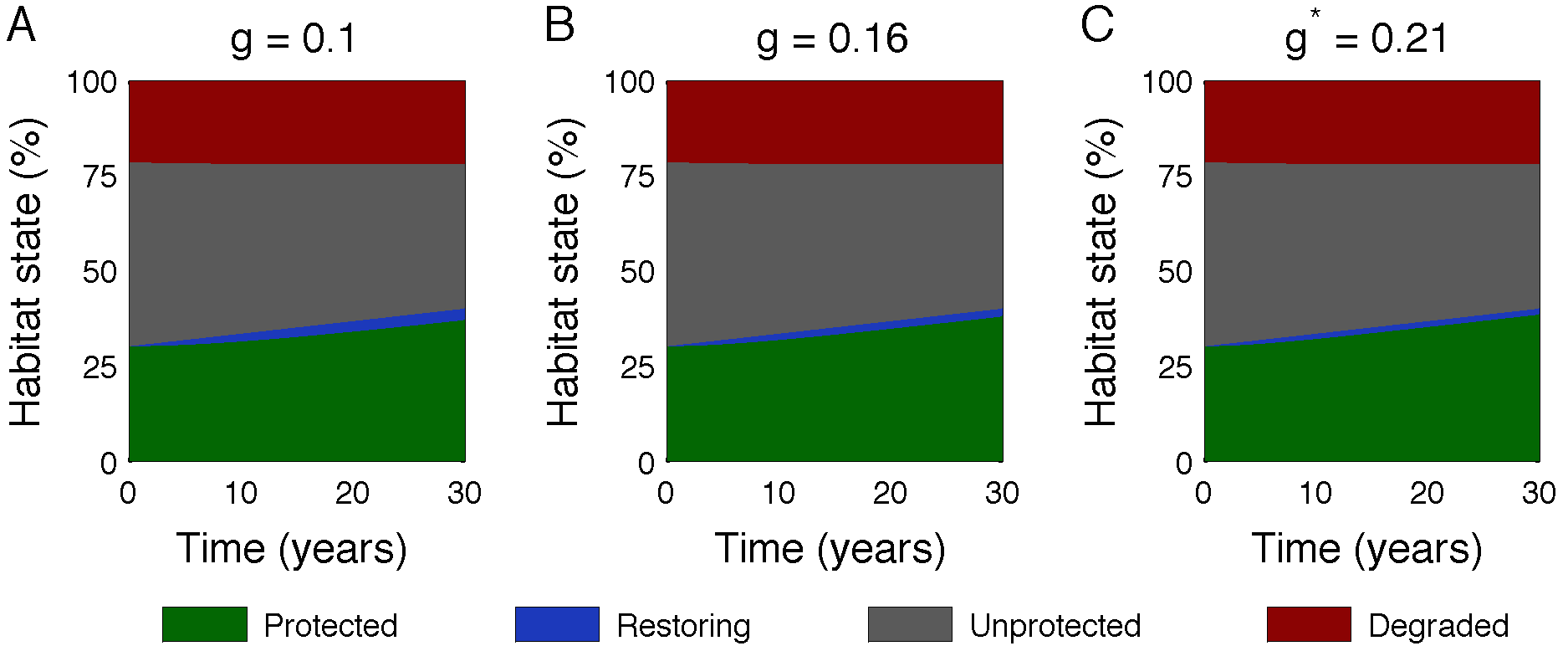

Supplement: S4 Fig — Results are shown for three different values of g, the rate of restoration. These are (A) the nominal estimate, g* = 0.21; (B) 75% of the nominal value, g = 0.16; and (C) 50% of the nominal value, g = 0.10. Optimal schedules are very similar, and in all cases give priority to restoration. Faster restoration rates simply mean that less habitat remains in the restoring state at any given time. The data used in this figure is given in S6 Data, and the Matlab code that generated it can be found in S2 Text. (TIF) [file pbio.1002052.s015.tif]

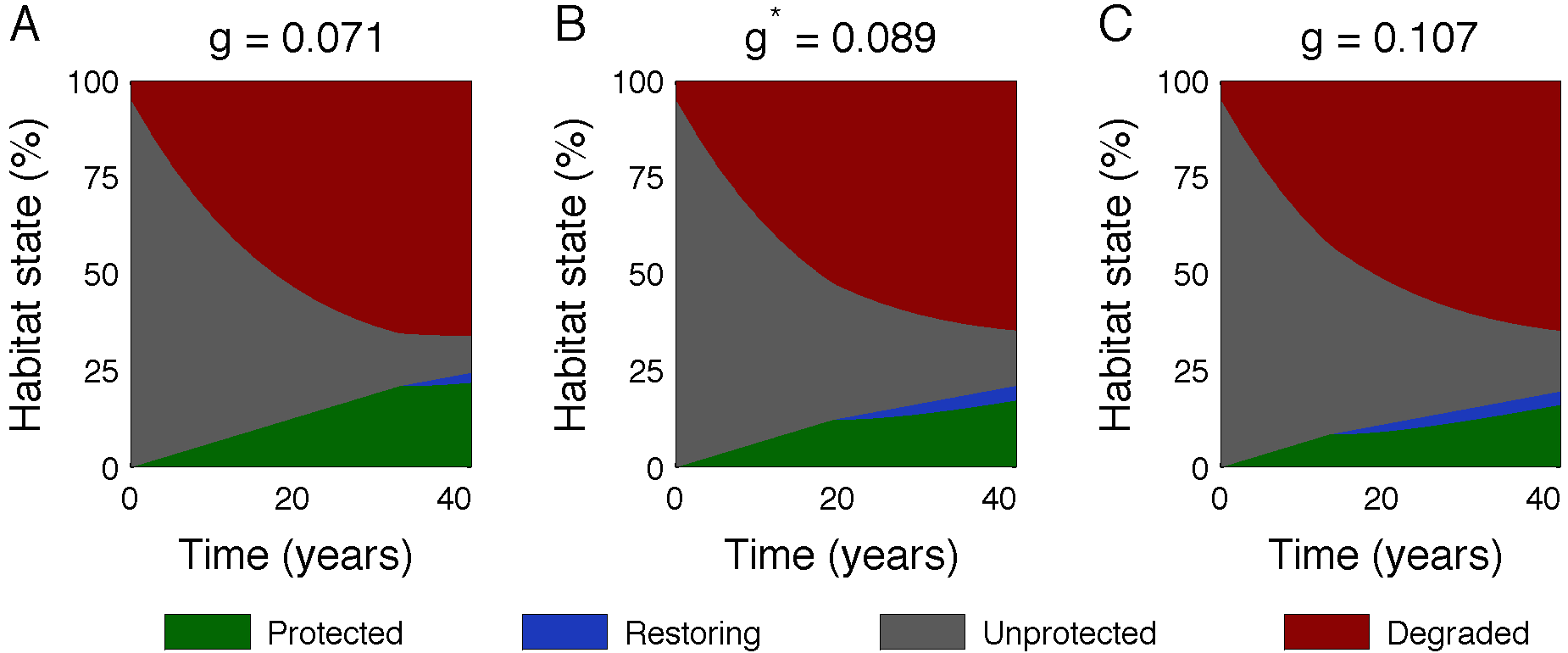

Supplement: S5 Fig — Results are shown for three different values of g, the rate of restoration. These are (A) the nominal estimate, g* = 0.089; (B) 80% of the nominal value, g = 0.071; and (C) 120% of the nominal value, g = 0.107. The optimal schedules are qualitatively similar, beginning with protection, then switching resources to restoration after years have elapsed. However, for lower values of g, managers should spend more time protecting intact habitat before they shift across to restoring degraded habitat. The data used in this figure is given in S7 Data, and the Matlab code that generated it can be found in S3 Text. (TIF) [file pbio.1002052.s016.tif]

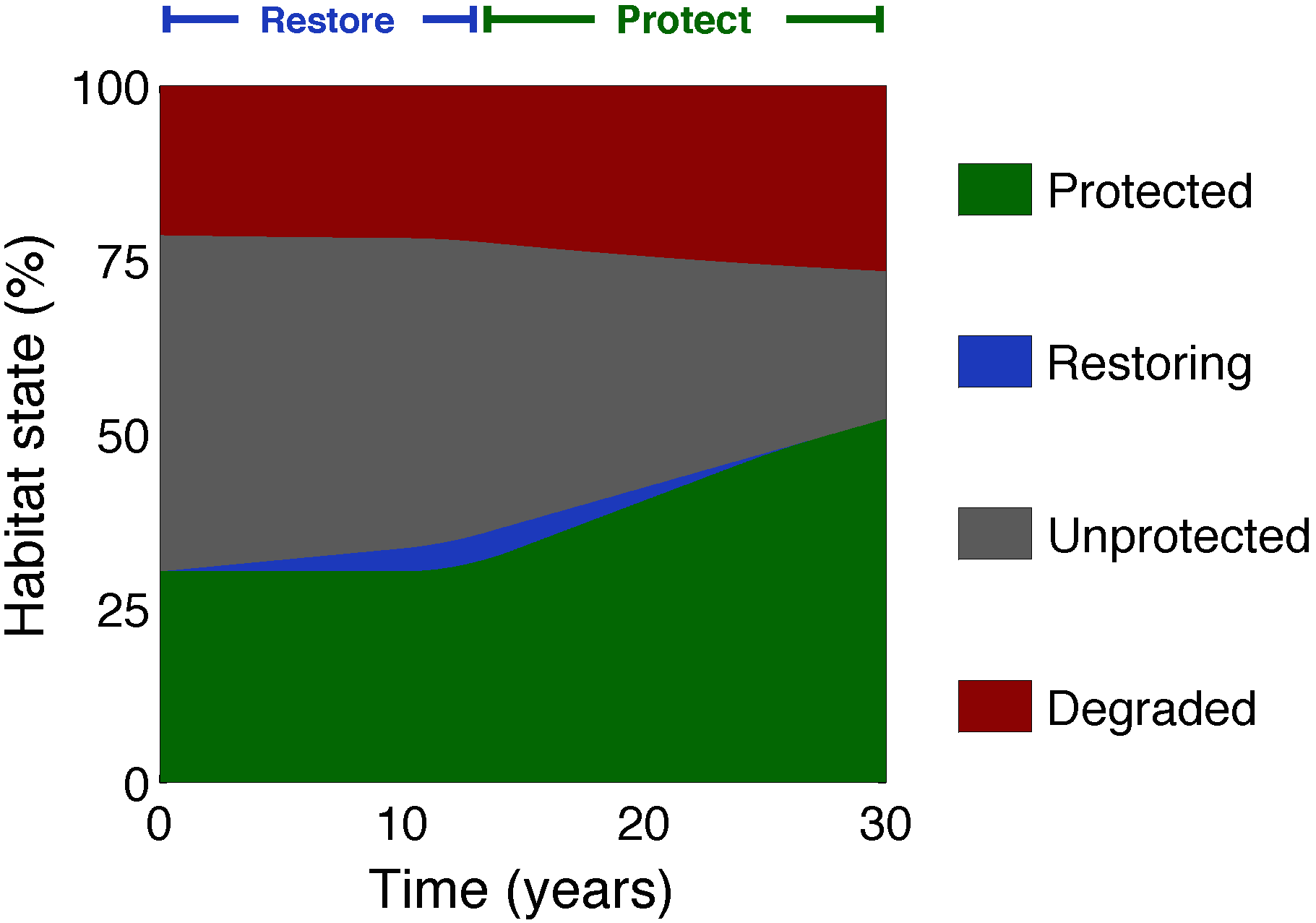

Supplement: S6 Fig — Resources are invested in restoration for approximately 13 years, after which time they are shifted across to protection for the remainder of the project duration. As with the continuous-restoration model, the schedule initially prioritises restoration. However, allocations differ in the latter years of the project, where optimisation of the fixed-delay model begins to protect land. This difference is partly due to the fact that any restoration in the last 14.5 years (the time lag of restoration) will produce no benefits since it will not be complete before the end of the project timeline. The data used in this figure is given in S8 Data, and the Matlab code that generated it can be found in S2 Text. (TIF) [file pbio.1002052.s017.tif]

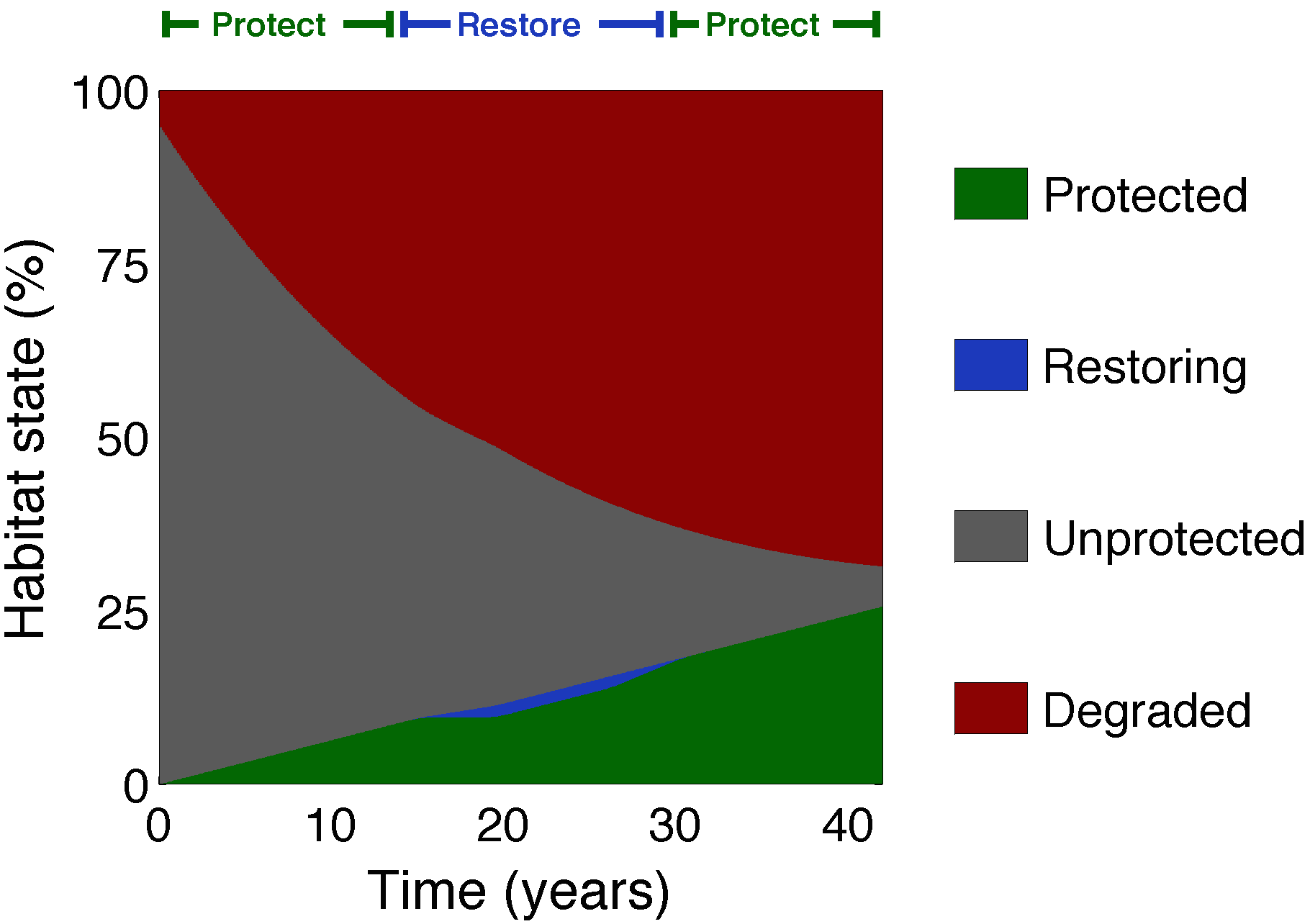

Supplement: S7 Fig — Resources are initially invested in protection for 15 years, before switching to restoration for another period of approximately 15 years. Resources are finally allocated back to protection for the final 12 years of the project. This spending pattern begins with the same sequence of allocations as the continuous-restoration model (i.e., protect-then-restore), before changing in the final years to prioritise protection. This difference reflects the fact that restoration undertaken in the final years of the project will be incomplete (and therefore of no value) when the project ends. The data used in this figure is given in S9 Data, and the Matlab code that generated it can be found in S3 Text. (TIF) [file pbio.1002052.s018.tif]

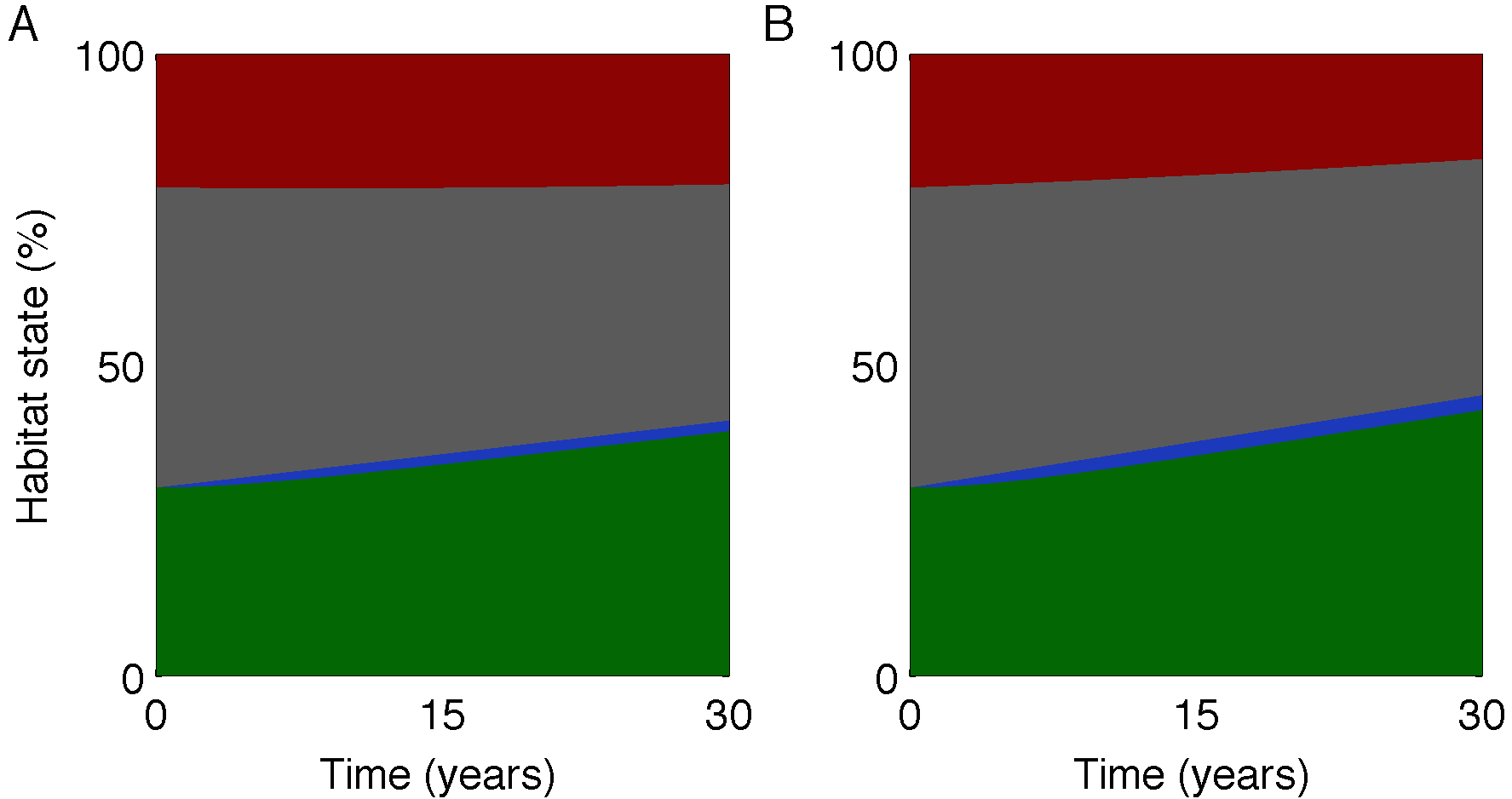

Supplement: S8 Fig — (A) When managers preferentially restore and protect less expensive land (i.e., mangrove habitat that is not suitable for aquaculture), and protection costs are therefore reduced by 25% from its nominal value. This also reduces the cost of restoration, since it also requires the purchase of land. (B) When managers preferentially restore abandoned land, there is therefore no opportunity cost for restoration (only the cost of the restoration action). This reduces the cost of restoration but leaves the cost of protection unchanged. Neither alternate assumption changes the qualitative conclusions of our analysis. The data used in this figure is given in S10 Data, and the Matlab code that generated it can be found in S2 Text. (TIF) [file pbio.1002052.s019.tif]

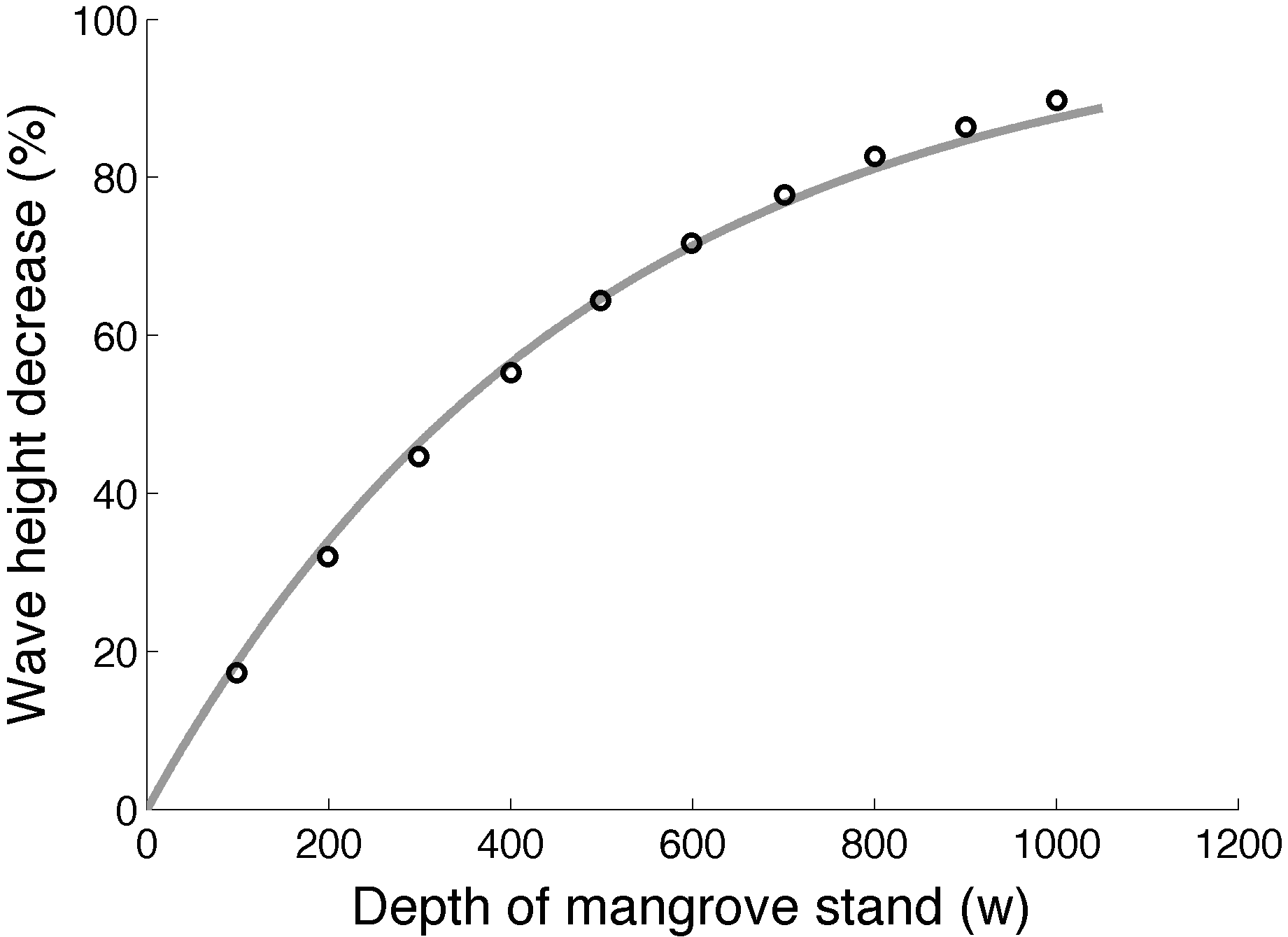

Supplement: S9 Fig — The data in this figure was sourced from the supplementary information in Barbier et al. [25], and is given in S11 Data. (TIF) [file pbio.1002052.s020.tif]
