# Supplementary material for: Optimal Conservation Outcomes Require Both Restoration and Protection
Source: PLoS Biol. 2015 Jan 27;13(1):e1002052. doi: 10.1371/journal.pbio.1002052 (PMC4308106; doi:10.1371/journal.pbio.1002052)
Supplement: S1 Text — (DOCX) [file pbio.1002052.s021.docx]

Supplementary information for “Optimal conservation outcomes require both restoration and protection”

Authors: Possingham HP, Bode M, & Klein CJ

**1 Dynamic conservation landscape model**

Habitat at time $t$ is classified as one of four states: intact and unprotected, $F(t)$, intact and protected, $P(t)$, cleared or degraded, $C(t)$, or undergoing restoration, $R(t)$, where $F(t)+C(t)+P(t)+R(t)=1$. Landscape dynamics are driven by management actions, and degradation driven by external actors. During a $T$-year project, managers allocate a varying proportion $0\leq u(t)\leq1$ of their fixed budget $(B)$ to protection, and the remainder$(1-u(t) )$to restoration. We assume that unprotected habitat is simultaneously being degraded at proportional rate $\delta$:

|  | $dF(t)/dt=-\delta F-uB/c_{P} ,$ $dP(t)/dt=uB/c_{P} +gR,$ $dC(t)/dt=\delta F-(1-u)B/c_{R} ,$ $dR(t)/dt=(1-u)B/c_{R} -gR,$ | Eq (S1) |
| --- | --- | --- |

where $g$ is the restoration rate, and $c_{P}$ and $c_{R}$ are the costs of protection and restoration respectively. For convenience, we may denote these state functions in vector form as $\tilde{x}\left( t \right)=\left[ F\left( t \right),P\left( t \right),C\left( t \right),R\left( t \right) \right]$. Note that the model describes changes in the landscape classification as instantaneous rates, although actual changes may occur through discrete changes (*e.g.*, the purchase of finite-sized land parcels), or with fixed time-lags (*e.g.*, landscape restoration). These dynamics are illustrated schematically in Figure S1, and their parameterisation is described in the sections following. However, we note that the central purpose of these examples is to illustrate the qualitative behaviour of optimal conservation decisions, not to prescribe exact conservation investment schedules. An application of these methods should be based on a more careful, site-specific parameterisation, and possibly a landscape model with more complex dynamics.

**1.1** **Landscape model parameterisation for south-east Asian mangrove forests**

Our primary conclusion in these analyses is that protection should not take automatic preference as a conservation action over restoration. As a result, when choosing parameters from uncertain ranges below, we have opted for values that bias our results away from restoration, to ensure our central conclusion is more robust. In the sections that follow, we apply parametric and structural sensitivity analyses for key parameters.

*1.1.1. Degradation rate:* $\delta$

We based our example on the upper estimate of the rate of decline of mangroves across Malaysia, which is estimated to have been between 0.5 – 0.8% annually over the last 30 years {Worldatlasofmangr:2010tu}, as specific values for our study region were not available. We therefore choose a value of $\delta=0.008$, but our results are qualitatively consistent within this range.

*1.1.2. Rate of mangrove restoration:* $g$

The restoration rate determines the delay between the beginning of a restoration project and the time at which restored habitat once again provides the same amount of ecosystem services as intact (i.e., never degraded) habitat. We are modelling coastal protection from mangrove forests, and so the restoration rate parameter needs to reflect the time required for actively restored mangroves forests to attenuate waves and reduce coastal damage.

We assume that mangrove forests only provide coastal protection once the trees are fully grown. Because smaller mangrove trees will still attenuate some wave energy, this assumption will bias our results away from the action of restoration. Our parameterisation is based on forestry data for *Sonneratia caseolaris*, a species that is native to Malaysia, and is commonly used for mangrove forest restoration (Leung & Tam 2013; Spalding *et al.* 2010). Studies show that this species grows at a rate of 0.38 +/- 0.15 centimetres per day, and has a maximum height of 20 meters (Spalding *et al.* 2010; Chen *et al.* 2012). Using stochastic simulations, this growth rate implies that 95% of newly planted seedlings (35 cm in height; Oxfam 2005) will reach their maximum height within 14.5 years. This estimate of restoration time agrees broadly with mangrove restoration projects which show the average individual of a vegetatively similar congeneric species (*Sonneratia apetala*) reaching 13.3 metres within 10 years, with a diameter at breast height (dbh) of 14.1 cm (Ren *et al*. 2009). *Sonneratia* forests of this age and size will provide substantial wave attenuation benefits to adjoining coastal regions. Mazda *et al.* (1997; 2006) report that 9-year old plantation of *Sonneratia* spp. (predominantly *S. caseolaris*, with an average dbh of 11.7 cm) can effectively attenuate the energy of severe waves, effecting a 50% reduction in their strength per 100 metres of forest width.

In our landscape model, habitat that is undergoing restoration becomes intact habitat at a proportional rate *g*. This means that if a parcel of land were purchased for restoration at time $t=0$, then over time the proportion remaining in the “restoring” state, $R\left( t \right)$, will decrease exponentially. The restoration rate *g* should match the reported growth rate data, and so we choose $g=0.21$, such that after 14.5 years (when 95% of the trees will have reached their maximum height), 95% of land purchased for restoration will have transitioned to intact habitat and will be providing coastal defence:

$0.05\approx\exp\left[ -0.21\times14.5 \right].$ Eq (S2)

Although our habitat model describes restoration as a continuous change, it would be better modelled with a fixed time-lag between the beginning of restoration and the realisation of intact habitat. In the sensitivity analyses below (§2.2), we relax this assumption and show that the optimal solution does not change qualitatively.

*1.1.3. Cost of protected area creation*: $c_{P}$

Mangrove forests are cleared for many reasons, but primarily for conversion to aquaculture and agriculture (Spalding *et al.* 2010). We based our cost of protection on the total net present value of the profit that would be forfeited by the aquaculture industry as a result of a protected area being created for the 30 year project. The annual profit of the four major brackish-water aquaculture systems in Malaysia range from USD $145,370 – 40,288,265 km^-2^ (Chong 2006). Because we are interested in showing that restoration can be the priority conservation action even under strong assumptions, we use the lower estimate in our model and do not include any ongoing management in the protected intact habitat. These choices will act to slightly bias our results away from restoration because they will make protection seem comparably inexpensive. At a discount rate of 3.22% (the long-term average US inflation rate), the endowed cost of 30 years of protection is $c_{P}=$ $2,841,300 km^-2^ (2006 USD).

*1.1.4. Cost of restoration actions:* $c_{R}$

The Sabah Forestry Department (2008) reported expenditure for mangrove restoration projects completed in 2006-2007, which includes all costs associated with restoring mangroves in Sabah. Annual restoration costs range from $172,000 – $289,400 km^-2^ (2006 USD). We again bias our results towards protection by assuming that restoration costs are at their upper bound, and assuming that, to undertake restoration actions, managers must create an endowment that can fund these annual costs for every year of the 30 year project. That is, managers must put aside the net present cost of the entire project in the first year. Assuming that land needs to be purchased for conservation prior to the onset of restoration activities, we add our estimate of the protection cost, given in §1.1.3, to the endowed costs of restoration. At a discount rate of 3.22% (the long-term average US inflation rate), the endowed cost of restoration is $c_{R}=$ $8,497,600 km^-2^ (2006 USD). Note that, while these two cost estimates have not been converted to 2014 dollars, they are from the same time period (2006-2007) and are therefore comparable.

*1.1.5. Initial Habitat distribution*

The current extent of mangrove forest in the study region is estimated to be 420 km^2^, consisting of 257.8 km^2^ of intact, unprotected habitat, and162.2 km^2^ of intact protected habitat. We assume that there is initially no ongoing restoration in Sabah. We can use decline rates to back-estimate the total extent of mangrove habitat in 1980, as this is when extensive mangrove clearing is likely to have begun in Malaysia (Spalding *et al.* 2010). The extent of mangroves in Sabah declined by 12% between 1980-1990 (Spalding *et al.* 1997), implying an annual loss rate that closely matches the change in mangrove habitat across Malaysia, which declined by 0.8% per year from 1990-2012 (Spalding et a. 2010). We therefore use this latter rate to back-estimate the original extent of intact mangrove habitat as 535 km^2^. These values create an initial habitat state distribution of:

$F\left( 0 \right)= 0.48; P\left( 0 \right)= 0.3;C\left( 0 \right)= 0.22; R\left( 0 \right)=0;$ Eq (S3)

**1.2 Landscape model parameterisation for the Atlantic Forests in Paraguay**

*1.2.1. Deforestation rates:* $\delta$

We based our example on a retrospective analysis of Paraguay's Atlantic Forest Ecoregion (PAFE), for which historical deforestation rates can be calculated by remote sensing. The PAFE is defined as the intersection between the WWF’s Atlantic Forest Ecoregion and the borders of Paraguay, a total area of 88,050 km^2^. This region of the Atlantic Forests was relatively intact until the early 1970s, coinciding with the launching of the Landsat program. Estimates of remaining forest cover (Huang *et al.* 2007) provide a relatively good fit to the exponential loss assumptions of our landscape model (Figure S2), and a best estimate of $\delta=0.04$.

*1.2.2. Rate of rainforest restoration:* $g$

Our rainforest restoration rate measures the rate at which restored rainforest recovers the same species richness as habitat that was never degraded. Our estimates are based on the research of Aide *et al.* (2000), who surveyed rainforests recolonised by passive restoration in 52 ex-pastures and forest sites in Luquillo and Carite, Puerto Rico. These sites had been abandoned for as long as 80 years, and which had previously been high diversity tropical forest. Aide *et al.* noted that the density, basal area, above-ground biomass and species richness of the recolonised rainforests had returned to levels similar to local old-growth forests within 40 years. We used an asymptotic exponential model (as proposed by our landscape model) to fit the reported species richness data, yielding regeneration rates of $g=0.052$ and $0.089$ for Carite and Luquillo respectively (Figure S3). We parameterised our PAFE model using $g=0.089$, on the assumption that active restoration could achieve results that were only as fast as rapid passive restoration.

It is important to note that we assume that restored habitat has returned to the intact state once it has regained its full suite of species. However, it is possible by this point that the species have returned, but not to their original abundances. Given that we assume the newly restored habitat will be reducing the extinction debt to the same extent as habitat that was never degraded, we are therefore ignoring the role that species abundances play in reducing extinction risk. While higher occupancy can reduce a species’ susceptibility to spatial catastrophes, abundance is an equally important factor. Unfortunately there is insufficient data on the rate at which abundance recovers to estimate rates, and we therefore perform a sensitivity analysis around the variable $g$ below (§2.1).

*1.2.3. Cost of protected area creation:* $c_{P}$

Our estimates of protected area purchase costs are based on cost-benefit estimates from the Amazonian rainforests in Brazil (Andersen 1997). Costs per square kilometer are based on the net present value (NPV) that would be forfeited as a result of protection, and would therefore be the minimum acceptable payment required to purchase the land for protection. We assume that candidate protected habitat would be presently forested, and would also be suitable for agriculture. Andersen’s calculations report that, in 1992, each cubic meter of timber yielded a once-off profit of $200, implying an average first year logging profit of $600 per hectare. If 63% of the land is then converted to pasture, 7% for annual crops and 2% for perennial crops, this implies a NPV of $89,000 km^-2^ (1992 USD). We correct this to 2014 USD using the long-term average US inflation rate of 3.22%. The result is an estimate of $c_{P}=$ $178,700 km^-2^ (2014 USD). We do not include any costs of ongoing management of the protected, intact habitat, an assumption that will act to bias our results towards protection.

*1.2.4. Cost of restoration actions:* $c_{R}$

Engel & Parrotta (2001) report establishment and ongoing maintenance costs for restoration operations based on direct seeding. The research restored tropical moist forests on three abandoned agricultural sites in south-eastern Brazil, using direct seeding with five early-successional Atlantic Forest species. We take the lower-value estimates of restoration costs using seed-planting (& ongoing management), $74,200 km^-2^ (2001 USD), and convert them into 2014 USD (again using the long-term inflation rate of 3.22%) giving $112,000. Added to the purchase price of logged land (*i.e.*, without timber values, since we are calculating the cost of restoring degraded habitat) from the previous section, giving an estimate of $c_{R}=$ $178,700 + $112,000 = $290,700 km^-2^ (2014 USD).

*1.2.5. Initial habitat distribution*

We initialise the habitat distribution in the PAFE based on the situation in approximately 1970. This decade saw the beginning of both rapid and extensive habitat degradation, as well as the creation of the country’s first protected areas (starting with the Parque Nacional Tinfunque in 1966). The initial forest landscape is predominantly intact but unprotected ($P\left( 0 \right)=0.95$), with minimal degradation/clearing ($C\left( 0 \right)=0.05$), and no ongoing restoration or existing protection ($R\left( 0 \right)=0$; $P\left( 0 \right)=0$).

**2 Sensitivity analyses of the model and parameterisation**

**2.1 Robustness of conclusions to restoration rate *g***

Although it is based on measured growth rates, our estimate of mangrove restoration rates in Sabah are quite rapid. Fortunately our key conclusion – that restoration should take preference over protection – is not sensitive to this parameter value. Figure S4 shows the optimal resource allocation schedule for three lower values of *g* (higher *g* values will only make restoration a higher priority), which are 75% and 50% of our nominal value. In all cases restoration should take preference over protection for the course of the project. It is only when the value of *g* falls to less than 25% of its estimated value that protection becomes the priority action (not shown).

Figure S5 shows similar robustness results for the Atlantic Forests in Paraguay, with the value of $g$ varying between $\pm20\%$ of our best estimate. Although in the Atlantic Forests example different restoration rates alter the timing and total spending on restoration, it remains optimal to invest in restoration before all available land has been protected.

**2.2 Robustness of conclusions to continuous restoration model**

The dynamic model of the restoration process (Eq. S1) assumes that land undergoing restoration is continuously transformed into intact habitat $(dR/dt=(1-u)B/c_{R} -gR)$. It would be more accurate to model the process of restoration as a fixed-time delay; that is, that land returns to the intact habitat state after a set number of years undergoing restoration. It is not clear how such a model could be incorporated into Pontryagin’s optimal control methdology, but the optimal allocation schedule can still be identified with reasonable accuracy using global optimisation techniques. Doing so indicates that the results from our two examples are valid for both continuous and delay models of restoration. We begin by altering our dynamic landscape model to incorporate time-delays:

$$dF/dt=-\delta F-u(t)B/c_{P}$$

$$dP/dt=u(t)B/c_{P} +(1-u(t-\tau))B/c_{R}$$

$$dC/dt=\delta F-(1-u(t-\tau))B/c_{R}$$

Eq (S4)

The “undergoing restoration” habitat state $\left( R \right)$ no longer requires an equation because restoration is modelled using the time delay $\tau$. In the mangrove forest example, we assume that this time delay is equal to the time taken for mangrove seedlings to reach maturity ($\tau=14.5$ years). In the Atlantic Forests, we choose the delay to be equal to the average time taken for forests to restore in the continuous model ($\tau=8$ years).

For both the Sabah mangrove ecosystem services example and the Atlantic forests biodiversity example, we use the Global optimisation package from Matlab (Package version 3.2.2; Matlab release 2012b) to identify the allocation schedule $u^{*}\left( t \right)$ that maximises the respective objective functions (Eq. 2 & 5 in the main text). We discretise the continuous control function into a series of 25 control variables $0\leq u_{i}\leq1$, which we choose at random, and allow the global optimiser to vary according to an internal search method. We distribute these discrete control points with equal spacing from $t=0,\ldots T$ and construct a continuous $u\left( t \right)$ from these values using linear interpolation (i.e., such that $u((i-1)T/24)=u\_i$). The optimiser chooses values for $u_{i}$ to maximise each objective function. To ensure that the method is identifying a single optima, we initialise the optimisation function from a large number of random starting search locations.

The results are shown in Figure S6 & Figure S7. Note that in both cases, habitat remains in the blue restoration state after restoration has ceased, reflecting the time-delay required before restored habitat can become intact and protected. The key results of our main analyses remain when restoration takes a fixed time period (Figure S5 & Figure S6). In the Sabah mangrove example, restoration should take precedence over protection; in the Atlantic Forests, restoration should begin before all available land has been protected. The fixed time delay has other impacts on the optimal allocation schedule. Most apparent is the return to protection in the final years of the time period. This switch mainly reflect the fact that, if restoration is not complete until a fixed time of $\tau$ years has elapsed, then any restoration in the final $\tau$ years of the project will not have time to accrue any benefits, and should not receive any investment.

**2.3 Robustness of conclusions to mangrove restoration costs**

Our parameterisation of the costs of mangrove protection and restoration were based on the value of land for aquaculture. However, some of the mangrove degradation in Sabah is for purposes of lower economic value than aquaculture, and this will incur lower opportunity costs. Further, some degraded habitat will be abandoned by aquaculture (Diana 2009), and its restoration will therefore not incur the opportunity costs of lost aquaculture profits. To test whether our results are robust to these cost assumptions, we calculate optimal allocations for two other cost scenarios: (1) managers preferentially restore and protect habitat that would have been used for less valuable economic uses, reducing the land acquisition price by 25%. This will affect both the restoration (from $8,497,600 km^-2^ to $7,787,300 km^-2^) and protection (from $2,841,300 km^-2^ to $2,130,900 km^-2^) costs, since restoration also incurs the opportunity costs; (2) managers protect land that would otherwise have been used for aquaculture, but only restore abandoned land. In the second example, managers incur no opportunity cost for restoration, leaving only the cost of the restoration activities (reducing the cost from $8,497,600 km^-2^ to $5,656,300 km^-2^). The cost of protection is unchanged at $2,841,300 km^-2^. Neither of these two changes had any qualitative impacts on the optimal allocation schedules (Figure S8).

**2.4 Alternative models of restoration**

Passive vs. active restoration

Our landscape model (Eq. S1) assumes that restoration is either all active, or all passive. However, once they purchase degraded habitat, managers have the option of either actively restoring that habitat, or allowing it to restore passively: each process occurs at a different rate, and incurs a different cost. This decision could be implemented into our framework by including an additional state variable: land undergoing passive restoration (*Q*), which is included separately from land undergoing active restoration (*R*):

|  | $dF(t)/dt=-\delta F-u_{P}B/c_{P} ,$ $dP(t)/dt=u_{P}B/c_{P} +gR+sQ,$ $dC(t)/dt=\delta F-u_{R}B/c_{R}-u_{Q}B/c_{Q} ,$ $dR(t)/dt=u_{R}B/c_{R} -gR,$  $dQ(t)/dt=u_{Q}B/c_{Q} -sQ,$  $u_{P}+u_{R}+u_{Q}=1.$ |
| --- | --- |

Eq (S5)

In this model active and passive restoration are implemented using similar functional forms. Habitat enters each state following a different management intervention, and becomes protected land after sufficient time has elapsed. The rate of passive restoration is slower than active restoration ($s<g$), but the cost of passive restoration is likely lower ($c_{Q}<c_{R}$). This model allows three decisions (protect, actively restore, passively restore), and so requires three proportional decision variables ($u_{P}$, $u_{R}$, and $u_{Q}$).

Restoration of abandoned land

Our landscape model also assumes that humans’ use of degraded habitat is ongoing, and that therefore there is no process of abandonment. If instead land is being abandoned, management must decide to either (1) spend resources on protection of intact habitat (the proportion $u_{P}$), or (2) implement active restoration on abandoned land that would otherwise undergo slower, passive restoration (the proportion $u_{A}$):

|  | $dF(t)/dt=-\delta F-u_{P}B/c_{P} ,$ $dP(t)/dt=u_{P}B/c_{P} +g\left( 1+u_{A}B/c_{A} \right)R,$ $dC(t)/dt=\delta F-hC ,$ $dR(t)/dt=hC -g\left( 1+u_{A}B/c_{A} \right)R,$  $u_{P}+u_{A}=1.$ |  |
| --- | --- | --- |

Eq (S6)

In this model, *h* defines the amount of abandonment in each timestep, which we assume is proportional to the amount of land that has been degraded (e.g., 2% of degraded habitat is abandoned in a given year, alternate functional forms for abandonment are obviously possible). Management spending can effectively increase the value of the restoration parameter $g$, with the variable $c_{A}$ describing the cost of accelerating the natural rate of passive restoration through active intervention.

Incomplete restoration

Our landscape model assumes that restored land will eventually regain the ecosystem attributes (e.g., biodiversity, or eosystem service provision) of “pristine” land (i.e., land that has never been degraded). If restored land cannot regain the full ecosystem attributes, at least within the timespan of the project, we can still compare restoration and protection with the addition of another state variable into the dynamic landscape model, and a reformulation of the objective function. For the ecosystem service provision objective, this new optimisation problem would be:

|  | $dF(t)/dt=-\delta F-uB/c\_P,$ $dP(t)/dt=uB/c\_P,$ $dC(t)/dt=\delta F-(1-u)B/c\_R,$  $dN(t)/dt=gR,$ $dR(t)/dt=(1-u)B/c\_R -gR.$  $\max_{0\leq u\left( t \right)\leq1} \int_{t=0}^{T} e^{-rt}\left[ 1-e^{-k\left( P\left( t \right)+F\left( t \right)+\phi N\left( t \right) \right)} \right]dt.$ |  |
| --- | --- | --- |

Eq (S7)

In this model, *N*(*t*) denotes land where restoration actions are complete. The variable $\phi$ represents the proportional degree to which the ecosystem service can be provided by completed restoration projects. Note that, compared to the original set of landscape equations, in this model the flow of restored land is from *R*(*t*) into the state *N*(*t*), rather than the state *P*(*t*).

While the first two alternate formulations of the landscape dynamics offer interesting alternatives that may be better suited to particular conservation problems, they will not affect our primary conclusions – that the protection of land should not automatically take precedence over restoration, and that restoration should sometimes begin before all available intact habitat has been protected. The presence of land abandonment, or the inclusion of passive restoration processes would both act to make restoration cheaper, and therefore *more* attractive as a conservation action. The final variation of the model, which allows for incomplete benefits from restored land, may affect the optimality of restoration if the performance of restored land (measured by the parameter $\phi$) is sufficiently less than one).

**3 Management objective functions**

The managers’ objectives are to maximise either the provision of ecosystem services, or minimise the extinction of species, by choosing an optimal allocation schedule $u\left( t \right)$. We relate $u\left( t \right)$ to both these objective functions through the dynamic landscape model in the following two sections.

**3.1 Management objective for ecosystem service provision**

In the mangrove example, managers attempt to maximise the provision of ecosystem services to coastal communities in Sabah, Malaysia. Barbier *et al.* (2008) calculated that coastal protection benefits accrue as a nonlinear function of the area of surrounding intact mangrove habitat. In their supplementary online information, the authors describe the proportional reduction $\rho$ in wave height that results from a mangrove forest of width $w$ as:

$$\rho\left( w \right)=8\times{10}^{-7}w^{2}+0.0016w+0.0128.$$

Eq (S8)

We chose to re-fit their data with an asymptotic exponential function for two reasons. First, the original quadratic relationship proposes an unrealistic shape (i.e., past a given width, Barbier *et al.*’s quadratic relationship implies that mangrove forests will increase wave height), and second, the positive y-intercept (0.0128) implies that no mangroves are needed for ecosystem benefits to accrue. The alternative functional form yields a similar fit to the data (Figure S9). We note that these results are for *Kandelia candel*, rather than *Sonneratia caseolaris*. While both were present in the Vietnamese mangrove plantation used in the study, the wave attenuation data was only available for *K. candel*. *S. caseolaris* will provide approximately 3 times greater wave attenuation than *K. candel* (Barbier *et al*. 2008), although this will not change our results if the proportional improvement is consistent.

By assuming that mangrove restoration creates a forest of uniform thickness in previously deforested areas along the Sabah coastline, we related the provision of coastal protection services to the protection and restoration of mangrove forests according to the equation:

|  | $E\left( t \right)=E_{0}\left[ 1-e^{-k\left( P\left( t \right)+F\left( t \right) \right)} \right],$ | Eq (S9) |
| --- | --- | --- |

where $P\left( t \right)$ is the area of protected mangrove forest, $F\left( t \right)$ is the area of unprotected but intact mangrove forest, and we use our best fit parameter: $k=2.1\times{10}^{-3}$. Note that this formula makes the conservative assumption that mangrove restoration $R\left( t \right)$does not provide coastal protection until it is fully restored. $E_{0}$ is the magnitude of the potential ecosystem services, but we nondimensionalise the objective function by measuring the proportion of pristine ecosystem services that are being provided:

|  | $E^{'}\left( t \right)=\frac{E\left( t \right)}{E_{0}}$ | Eq (S10) |
| --- | --- | --- |

This normalised objective function allows us to measure ecosystem service provision as the proportional reduction in damage caused by a lower wave height in areas sheltered by mangrove forests. Over the course of a $T$year project, managers choose to allocate their conservation budget between protection and restoration to maximize:

|  | $\max_{u\left( t \right)} \int_{t=0}^{T} e^{-rt}\left[ 1-e^{-k\left( P\left( t \right)+F\left( t \right) \right)} \right]dt,$ | Eq (S11) |
| --- | --- | --- |

where $r$ is the discount rate of future benefits relative to present benefits.

**3.2 Management objective for biodiversity conservation**

The managers’ objective in conserving rainforest in the PAFE is to minimize the number of extinctions in that region during the course of a $T$-year project. Extinctions in the Atlantic Forests will occur by the creation and subsequent relaxation of extinction debts (Brooks and Balmford 1996). Extinction debts occur when, after habitat degradation, the number of extant species exceeds the equilibrium number that can be supported by the remaining habitat. The resulting rate of species extinction is modelled as proportional to the size of the species debt: the difference between the extant species richness and the number of species supported at equilibrium (Ferraz *et al.* 2003). We incorporate the additional dynamics of species extinction into the dynamic landscape model with the addition of another dynamic state variable, the rate of change of the number of extant species, $S\left( t \right)$:

|  | $\frac{dS\left( t \right)}{dt}=\theta\left[ S\left( t \right)-S^{*} \right]=\theta\left[ S\left( t \right)-\alpha\left( P\left( t \right)+F\left( t \right) \right)^{z} \right],$ | Eq (S12) |
| --- | --- | --- |

where $\theta$ is the extinction debt relaxation rate. Brooks *et al*. (1999) estimate the time-lag between deforestation and extinction using bird species in tropical forest fragments in east Africa. For fragments of approximately 1000 hectares, they estimate that the half-life of extinctions (i.e., the time it takes for half of the expected extinctions to occur) ranges between 23 and 80 years. We use the lower estimate of 23 years, implying that $\theta=-0.03$. For bird species in tropical rainforests, we estimate $z=0.18$ based on the results of Diamond’s (1975) studies of island birds in New Guinea’s Dampier straits. Although this value is far from biogeographically ideal, we note that dynamic allocation schedules are generally robust to this value (Bode and Murdoch 2009). To avoid choosing a particular value for $\alpha$, we express species as a proportion of the total diversity:

|  | $s\left( t \right)=\frac{S\left( t \right)}{\alpha}$ | Eq (S13) |
| --- | --- | --- |

allowing us to present our results as the proportion of the original number of bird species in the region that are still extant. Managers will ensure the fewest species extinctions over a *T*-year project if they choose an allocation schedule $u\left( t \right)$ that satisfies:

|  | $\min_{u\left( t \right)} \int_{t=0}^{T} \left[ s\left( t \right)-\left( P\left( t \right)+F\left( t \right) \right)^{z} \right]dt,$ | Eq (S14) |
| --- | --- | --- |
|  |  |  |

where the dynamics of $F\left( t \right)$and $P\left( t \right)$are governed by Eq (S1).

**4. Calculating the optimal allocation schedule**

The optimal allocation schedule can be identified by applying Pontryagin’s maximum principle to the system dynamics in Eq (S1), and each problem’s objective function (Eq S11 and Eq S14). We note at this point that the proportion of degraded habitat (a variable not present in either objective functions) is $C\left( t \right)=1-P\left( t \right)-F\left( t \right)-R\left( t \right)$, and can therefore be considered a dynamic constraint, rather than a state variable.

**4.1 Optimal allocation schedule for ecosystem service provision**

To define the optimisation Hamiltonian for ecosystem service provision, $H_{E}\left( t,\tilde{x},u,\tilde{\lambda} \right)$, we first create piecewise differentiable “costate” functions that correspond to the three key state functions:

| $\frac{d\lambda_{P}\left( t \right)}{dt}=-\frac{\partial H}{\partial P},$ | $\frac{d\lambda_{R}\left( t \right)}{dt}=-\frac{\partial H}{\partial R}.$ | Eq (S15) |
| --- | --- | --- |
| $\frac{d\lambda_{F}\left( t \right)}{dt}=-\frac{\partial H}{\partial F},$ |  |  |

Along with the management objective (Eq S11), these functions create the ecosystem services Hamiltonian:

|  | $H_{E}=\left[ 1-e^{-k\left( P\left( t \right)+F\left( t \right) \right)} \right]e^{-rt}+\lambda_{F}\frac{dF}{dt}+\lambda_{R}\frac{dR}{dt}+\lambda_{P}\frac{dP}{dt},$ | Eq (S16) |
| --- | --- | --- |

The optimal budget allocation schedule $u^{*}(t)$ is chosen by maximizing the Hamiltonian at each point in time (Lenhart and Workman 2007):

|  | $H_{E}\left( t,\tilde{x}^{*},u,\tilde{\lambda} \right)\geq H_{E}\left( t,\tilde{x}^{*},u^{*},\tilde{\lambda} \right).$ | Eq (S17) |
| --- | --- | --- |

Substituting the state variables into Eq (S16) and rearranging reveals a Hamiltonian that is linearly dependent on the control variable $u\left( t \right)$:

|  | $H_{E}=\left[ 1-e^{-k\left( P\left( t \right)+F\left( t \right) \right)} \right]e^{-rt}-\lambda_{F}\left( \delta F+\frac{uB}{c_{P}} \right)+\lambda_{R}\left( \frac{\left( 1-u \right)B}{c_{R}}-gR \right)+\lambda_{P}\left( \frac{uB}{c_{P}}+gR \right)$,  $H_{E}=uB\left[ \frac{\lambda_{P}-\lambda_{F}}{c_{P}}-\frac{\lambda_{R}}{c_{R}} \right]+\left[ e^{-rt}\left( 1-e^{-k\left( A+R \right)} \right)-\delta F\lambda_{F}+\left( \frac{B}{c_{R}}-gR \right)\lambda_{R}+gR\lambda_{P} \right]$.  $H_{E}=uB\sigma\left( t \right)+\phi_{E}\left( t \right)$ | Eq (S18) |
| --- | --- | --- |

This implies that the optimal control path is “bang-bang”, and at any point in time, resources should be allocated either to protection or restoration, not a combination of both. Note that in Eq (S18), the gradient of the linear Hamiltonian, and therefore the optimal allocation of resources, depends on the “switching function”:

|  | $\sigma\left( t \right)=\frac{\lambda_{P}\left( t \right)-\lambda_{F}\left( t \right)}{c_{P}}-\frac{\lambda_{R}\left( t \right)}{c_{R}}.$ | Eq (S19) |
| --- | --- | --- |

This function has a relatively straightforward interpretation. The first term reports the per-dollar shadow value (i.e., the expected future value assuming optimal behaviour) of converting land from intact but unprotected, to intact and protected. The second term reports the per-dollar shadow value of beginning the process of restoration instead (these costate values are themselves complex functions). Predictably, if the shadow value of protection is greater than that of restoration, funding should be preferentially invested in protection. We note that some linear Hamiltonians allow partial allocations during a singular arc, when the switching function is equal to zero over a finite time period. We assume in this case that this would not happen, since it would require the three costate variables to each change through time in such a way that Eq (S19) would remain zero. This seems very unlikely, particularly given that the functional form of these variables will be different, since they occur in the Hamiltonian in different ways. We verify the bang-bang form of the optimal solution using Matlab’s numerical global optimisation method described above in §2.2.

We identify the optimal control path by applying the Forward-Backward Sweep method developed by Hackbusch (1978), and described in Lenhart & Workman (2007), and using the transversality (boundary) conditions that correspond to a zero scrap value for intact habitat, and habitat undergoing restoration, at the terminal time:

|  | $\lambda_{F}\left( T \right)=\lambda_{R}\left( T \right)=\lambda_{P}\left( T \right)=0$ | Eq (S20) |
| --- | --- | --- |
|  |  |  |

**4.2 Optimal allocation schedule for biodiversity conservation**

We define the optimisation Hamiltonian for biodiversity conservation, $H_{B}\left( t,\tilde{x},u,\tilde{\lambda} \right)$, in much the same way as for ecosystem service provision, with the added complication of an additional state variable for the change in the number of extant species resulting from the rate of extinction debt relaxation (Eq S12). This in turn generates a costate equation corresponding to $S\left( t \right)$, in addition to those of the habitat state variables (Eq S15).

|  | $\frac{d\lambda_{S}\left( t \right)}{dt}=-\frac{\partial H}{\partial S}.$ | Eq (S21) |
| --- | --- | --- |

The Hamiltonian is a combination of the biodiversity management objective (Eq S14) and the costate variables:

|  | $H_{B}=S\left( t \right)-\left( P\left( t \right)+F\left( t \right) \right)^{z}+\lambda_{F}\frac{dF}{dt}+\lambda_{R}\frac{dR}{dt}+\lambda_{P}\frac{dP}{dt}+\lambda_{S}\frac{dS}{dt}$, | Eq (S22) |
| --- | --- | --- |

which is another linear function of the control variable, $u\left( t \right)$:

|  | $H_{B}=uB\left[ \frac{\lambda_{P}-\lambda_{F}}{c_{P}}-\frac{\lambda_{R}}{c_{R}} \right]-\left[ \theta\left( 1-\lambda_{S} \right)\left( S-\left( F+P \right)^{z} \right)-\lambda_{F}\delta F+\lambda_{R}\left( \frac{B}{c_{R}}-gR \right)+\lambda_{P}gR \right]$,  $H_{B}=uB\sigma\left( t \right)+\phi_{B}\left( t \right)$ | Eq (S23) |
| --- | --- | --- |

That is, with the same switching function $\sigma\left( t \right)$ identified in the ecosystem services example (although constructed with different costate functions). Because the management objective is to minimise extinctions over the course of a finite management period, we once again assign no scrap value for any of the state variables.

|  | $\lambda_{F}\left( T \right)=\lambda_{R}\left( T \right)=\lambda_{P}\left( T \right)=\lambda_{S}\left( T \right)=0$ | Eq (S24) |
| --- | --- | --- |

The optimal control schedule is again constructed using Lenhart & Workman’s (2007) sweep methods.

**5 Derivation of the myopic heuristic**

We derive a heuristic decision rule via a simple temporal discretisation and linearisation of the landscape dynamics in Eq (S1). The initial distribution of land is $\tilde{x}_{0}=\left[ F\left( 0 \right)=F_{0},R\left( 0 \right)= R_{0},P\left( 0 \right)= P_{0},C\left( 0 \right)= C_{0} \right]$. We also include the possibilities that restoration or protection actions are unsuccessful, events that occur with probability $\left( 1-q_{P} \right)$ and $\left( 1-q_{R} \right)$.

Regardless of the specific benefit function (i.e., ecosystem service provision or biodiversity conservation), if the managers spend all their resources on protection, then the land distribution at $t=1$ will be:

| $F\left( 1 \right)=\left( 1-\delta\right)\left( F_{0}-\frac{q_{P}B}{c_{P}} \right),$ | $R\left( 1 \right)=R_{0}\left( 1-g \right),$ | Eq (S25) |
| --- | --- | --- |
| $P\left( 1 \right)=P_{0}+gR_{0}+\frac{q_{P}B}{c_{P}},$ | $C\left( 1 \right)=C_{0}+\delta\left( F_{0}-\frac{q_{P}B}{c_{P}} \right).$ |  |

If there is no further investment, at $t=2$ the land state that results from the initial decision to protect becomes:

| $F\left( 2 \right)=\left( 1-\delta\right)^{2}\left( F_{0}-\frac{q_{P}B}{c_{P}} \right),$ | $R\left( 2 \right)=R_{0}\left( 1-g \right)^{2},$ | Eq (S26) |
| --- | --- | --- |
| $P\left( 2 \right)=P_{0}+gR_{0}\left( 2-g \right)+\frac{q_{P}B}{c_{P}},$ | $C\left( 2 \right)=C_{0}+\left( 2-\delta\right)\left( F_{0}-\frac{q_{P}B}{c_{P}} \right).$ |  |

Alternatively, managers could spend the money restoring land, in which case the land distribution at $t=1$ will be:

| $F\left( 1 \right)=\left( 1-\delta\right)F_{0},$ | $R\left( 1 \right)=R_{0}\left( 1-g \right)+\frac{q_{R}B}{c_{R}},$ | Eq (S27) |
| --- | --- | --- |
| $P\left( 1 \right)=P_{0}+gR_{0}$ | $C\left( 1 \right)=C_{0}+\delta F_{0}-\frac{q_{R}B}{c_{R}}.$ |  |

At $t=2$, the habitat distribution that results from the initial decision to protect becomes:

| $F\left( 2 \right)=\left( 1-\delta\right)^{2}F_{0},$ | $R\left( 2 \right)=R_{0}\left( 1-g \right)^{2}+\frac{\left( 1-g \right)q_{R}B}{c_{R}},$ | Eq (S28) |
| --- | --- | --- |
| $P\left( 2 \right)=P_{0}+g\left( R_{0}\left( 2-g \right)+\frac{q_{R}B}{c_{R}} \right),$ | $C\left( 2 \right)=C_{0}+\delta F_{0}\left( 2-\delta\right)-\frac{q_{R}B}{c_{R}}.$ |  |

Because both the biodiversity conservation and ecosystem service provision functions are monotonic in $\left( F\left( t \right)+P\left( t \right) \right)$, maximising the expected benefit is equivalent to maximising $\left( F\left( 2 \right)+P\left( 2 \right) \right)$. As a result, in the short term we would prioritise the protection of intact habitat if:

|  | $-\left( 1-\delta\right)^{2}\frac{q_{P}B}{c_{P}}+gR_{0}\left( 2-g \right)+\frac{q_{P}B}{c_{P}}>+g\left( R_{0}\left( 2-g \right)+\frac{q_{R}B}{c_{R}} \right)$  $\Longrightarrow\frac{q_{R}g}{q_{P}\delta\left( 2-\delta\right)}<\frac{c_{R}}{c_{P}}$ | Eq (S29) |
| --- | --- | --- |

If we assume that $\delta$ is relatively small, we can ignore the effects of any $\delta^{2}$ terms, simplifying the condition to:

|  | $\frac{g}{2\delta}<\frac{c_{R}/q_{R}}{c_{P}/q_{P}}$ | Eq (S30) |
| --- | --- | --- |

***For restoration to be prioritised over protection, the relative speed of restoration to protection has to be greater than twice their relative cost.***

If the probability of failure of both actions is the same ($q_{R}=q_{P}$), restoration is preferred when its higher costs ($c_{R}/c_{P}$) are outweighed by its superior ability to reduce the net loss of intact habitat (expressible as $g/2\delta$):

|  | $\frac{g}{2\delta}<\frac{c_{R}}{c_{P}}$ | Eq (S31) |
| --- | --- | --- |

For example, if the cost of restoration is twice the cost of protection, the rate of restoration should be four times as fast as the rate of habitat loss. However, because both restoration and protection will sometimes be unsuccessful; the probability of failure can be consider equivalent to either (1) a decrease in the rate of both conservation processes, or (2) an increase in their implementation costs.

**6 References cited**

1. Aide T. M., Zimmerman J. K., Pascarella J. B., Rivera L. & Marcano-Vega H. (2000) Forest regeneration in a chronosequence of tropical abandoned pastures: implications for restoration ecology. *Restoration Ecology* **8**, 328-38.
2. Andersen L. E. (1997) A Cost-Benefit Analysis of Deforestation in the Brazilian Amazon. Texto para discussão No 455, Institudo de Pesquisa Economica Aplicada, Rio de Janeiro.
3. Barbier, E. B. *et al.* (2008) Coastal ecosystem-based management with nonlinear ecological functions and values. *Science* **319**, 321-323, doi:DOI 10.1126/science.1150349.
4. Bode M. & Murdoch W. (2009) Cost-effective conservation decisions are robust to uncertainty in the species-area relationship. *Proceedings of the National Academy of Sciences USA* **106**, E12; author reply E3.
5. Brooks T. M. & Balmford A. (1996) Atlantic forest extinctions. *Nature* **380**, 115.
6. Brooks T. M., Pimm S. L. & Oyugi J. O. (1999) Time lag between deforestation and bird extinction in tropical forest fragments. *Conservation Biology* **13**, 1140-50.
7. Chen L., Zeng Z., Tam N., Lu W., Luo Z., Du X., Wang J.(2012) Comparing carbon sequestration and stand structure of monoculture and mixed mangrove plantations of Sonneratia caseolaris and S. apetala in Southern China. *Forest Ecology and Management* **284,** 222–229.
8. Chong, V. C. (2006). Sustainable utilization and management of mangrove ecosystems of Malaysia, *Aquatic Ecosystem Health and Managment* **9**, 249-260.
9. Diamond J. M. (1975) The island dilemma: lessons of modern biogeographic studies for the design of natural reserves. *Biological Conservation* **7**, 129-46.
10. Diana J. S. (2009) Aquaculture production and biodiversity conservation. *Bioscience* **59**, 27-38.
11. Engel V. L. & Parrotta J. A. (2001) An evaluation of direct seeding of reforestation of degraded lands in central Sao Paulo state, Brazil. *Forest Ecology and Management* **152**, 169-81.
12. Ferraz G., Russell G. J., Stouffer P. C., Bierregaard R. O., Pimm S. L. & Lovejoy T. E. (2003) Rates of species loss from Amazonian forest fragments. *Proceedings of the National Academy of Sciences USA* **100**, 14069-73.
13. Hackbusch W (1978) A numerical method for solving parabolic equations with opposite orientations. *Computing* **20**, 229-240.
14. Huang C., Kim S., Altstatt A., Townshend J. R. G., Davis P., Song K., Tucker C. J., Rodas O., Yanosky A., Clay R. & Musinsky J. (2007) Rapid loss of Paraguay's Atlantic forest and the status of protected areas - A Landsat assessment *Remote Sensing of Environment* **106**, 460-6.
15. Lenhart S. & Workman J. T. (2007) *Optimal control applied to biological models*. Chapman & Hall, Boca Raton.
16. Leung J., Tam N. (2013) Influence of plantation of an exotic mangrove species,*Sonneratia caseolaris* (L.) Engl., on macrobenthic infaunal community in Futian Mangrove National Nature Reserve, China. *Journal of Experimental Marine Biology and Ecology* **448**, 1-9.
17. Mazda Y., Magi M., Ikeda Y., Kurokawa T., Asano T. (2006) Wave reduction in a mangrove forest dominated by *Sonneratia* sp. *Wetlands Ecology and Management* **14**, 365-378.
18. Mazda Y., Magi M., Kogo M., Nguyen Hong P. (1997) Mangroves as a coastal protection from waves in the Tong King delta, Vietnam. *Mangroves and Salt Marshes* **1**, 127-135.
19. Oxfam (2005) *Best practice guidelines on restoration of mangroves in Tsunami affected areas*. Downloaded on 12/03/2014 from: south-asia.wetlands.org/LIBRARY/tabid/645/mod/1570/articleType/ArticleView/articleId/2183/Default.aspx.
20. Ren H., Chen H., Li Z., Han W. (2009) Biomass accumulation and carbon storage of four different aged *Sonneratia apetala*plantations in Southern China. *Plant and Soil* **327,** 279-291.
21. Sabah Forestry Department. (2008) Mangrove forest managment and restoration. Sabah Forestry Department, Kota Kinabalu.
22. Spalding M., Blasco, F., Field, C. (1997) *World Atlas of Mangroves*. International Society for Mangrove Ecosystems, WCMC, National Council for Scientific Research, Paris.
23. Spalding M., Kainuma M. & Collins L. (2010) *World Atlas of Mangroves*. Earthscan, London.
24. World Wildlife Fund Malaysia. Mangrove extent for Tun Mustapha Park. Obtained from WWF-Malaysia in May 2011.
