# Supplementary material for: Optimal Conservation Outcomes Require Both Restoration and Protection
Source: PLoS Biol. 2015 Jan 27;13(1):e1002052. doi: 10.1371/journal.pbio.1002052 (PMC4308106; doi:10.1371/journal.pbio.1002052)
Supplement: S3 Text — (DOCX) [file pbio.1002052.s023.docx]

function Figure_3()

% Define the model parameters

TotalOriginalExtent = 88050; % Total original extent of the PAFE

IntactAndUnprotected = 0.95*TotalOriginalExtent; % Amount remaining intact and unprotected

IntactAndReserved = 0.0*TotalOriginalExtent; % Amount that is reserved and intact

DR = 0.0322; % Discount rate

AcquisitionCostPerKM2 = 89000*(1+DR)^(2014-1992); % Cost of acquisition

RevegetationCostPerKM2 = 89000*(1+DR)^(2014-1992) + 74200*(1+DR)^(2014-2001); % Cost of revegetation

B = 100e6; % Total annual budget

Alpha = 45; z = 0.18; % SPAR benefit function

A_0 = IntactAndUnprotected/TotalOriginalExtent; % Available land proportion

R_0 = IntactAndReserved/TotalOriginalExtent; % Reserved land proportion

S_0 = Alpha*1; % Initial number of species

V_0 = 0; % Revegetating land proportion

L_0 = 1 - A_0 - R_0 - V_0; % Degraded land proportion

Cp = AcquisitionCostPerKM2*TotalOriginalExtent; % Cost of reservation

Cv = RevegetationCostPerKM2*TotalOriginalExtent; % Cost of revegetation + reservation

d = 0.04; % Land loss rate

g = 0.089; % Revegetation rate

theta = 0.03; % Extinction debt relaxation rate

T = 42; % Length of the project

dt = 0.1; % Simulation timestep size

% Calculate and output the rule of thumb for this example

disp('============'), disp('g / d'), ROT_LHS = g/2/d

disp('2 * Cv / Cp'), ROT_RHS = Cv/Cp

if ROT_LHS < ROT_RHS, disp('Protect first'),

else, disp('Restore first'), end, disp('============')

% Costate variables have to be initialised

LA_0 = 4.29531815; LR_0 = 11.8137815; LV_0 = 10.1375179; LS = exp(theta*([dt:dt:T] - T));

% This loop continues to change the shooting method attempts until the costate

% variables match their terminal conditions.

Diff = 1; count = 1;

while Diff > 1e-4

% Calculate the terminal values of the costate equations with the

% current initial conditions, using the function SubOC_Mangroves (see below)

[LA,LR,LV,LS,u,Spp,Af,Rf,Vf,Lf] = SubOC_AtlanticF(LA_0,LR_0,LV_0,LS,Alpha,S_0,z,theta,Cp,Cv,B,A_0,R_0,V_0,L_0,d,g,T,dt);

% This difference indicates how far we are from satisfying the

% optimality conditions

Diff = abs(LA(end))+abs(LV(end))+abs(LR(end)); DEL = 0.02; count = count+1;

% Calculate new initial conditions for the costate variables based on

% how far from the correct terminal values they are, and what direction

% the error is in.

LA_0 = LA_0 - DEL.*LA(end); LR_0 = LR_0 - DEL.*LR(end); LV_0 = LV_0 - DEL.*LV(end);

% Output the progress of the search

if mod(count,1) == 1, disp([LA(end) LR(end) LV(end) LS(end)]), end

% If the difference is increasing, return an error.

if Diff > 1e8, disp('ERROR'); return; end

end

% Plot the optimal results

Plotting_Routine(u,Cp,Cv,B,S_0,Alpha,z,theta,A_0,R_0,V_0,L_0,Spp,d,g,dt,T,LA,LV,LR,LS);

% ==================== SUBROUTINES ====================

function [LA,LR,LV,LS,u,S,A,R,V,L] = SubOC_AtlanticF(LA,LR,LV,LS,Alpha,S_0,z,theta,Cp,Cv,B,A,R,V,L,d,g,T,dt)

% This function is used to solve for the optimal allocation strategy. It

% takes in initial conditions for both the state and costate variables (the

% initial conditions for the costate variables have been chosen via the

% shooting method, the initial conditions for the state variables are

% known), and forward simulates them both (the equations are coupled) to

% generate the terminal values of the costate variables. These are passed

% back to the main function, which is attempting to match them to the known

% transversality conditions.

bp = B/Cp; bv = B/Cv; S(1) = S_0;

% Forward simulate the state and costate variables

for i = 1:T./dt-1

% Calculate the switching function and determine the optimal control

SF(i) = bp*(LR(i)-LA(i))-bv*LV(i);

if SF(i) > 0, u(i) = 1; else u(i) = 0; end

if A(i) <= 0; u(i) = 0; end; if L(i) <= 0; u(i) = 1; end;

% Calculate and implement the land changes

Protected = min([A(i),dt*u(i)*bp]);

Degraded = dt*d*A(i);

Revegetated = min([L(i),dt*(1-u(i))*bv]);

Recovered = dt*g*V(i);

SppLost = dt*theta*(S(i) - Alpha*(A(i)+R(i))^z);

if SppLost < 0, SppLost = 0; end

A(i+1) = A(i) - Degraded - Protected;

R(i+1) = R(i) + Protected + Recovered;

L(i+1) = L(i) + Degraded - Revegetated;

V(i+1) = V(i) + Revegetated - Recovered;

S(i+1) = S(i) - SppLost;

% Iterate the costate variables

LA(i+1) = LA(i) + dt*(d*LA(i) - LS(i)*theta*z*Alpha*(A(i)+R(i))^(z-1));

LR(i+1) = LR(i) + dt*(-LS(i)*theta*z*Alpha*(A(i)+R(i))^(z-1));

LV(i+1) = LV(i) + dt*(g*(LV(i)-LR(i)));

end

function [A,R,V,L,S] = SubForce_AtlanticF(u,Alpha,S_0,z,theta,Cp,Cv,B,A,R,V,L,d,g,T,dt);

% This function forward simulates the state variables given a particular

% control function, passed as the vector u. It then calculates the

% resulting state variables values along the project timeline and returns

% them

bp = B/Cp; bv = B/Cv; S(1) = S_0;

% Forward simulate the state variables

for i = 1:T./dt-1

% Calculate and implement the land changes

Protected = min([A(i),dt*u(i)*bp]);

Degraded = dt*d*A(i);

Revegetated = min([L(i),dt*(1-u(i))*bv]);

Recovered = dt*g*V(i);

SppLost = dt*theta*(S(i) - Alpha*(A(i)+R(i))^z);

% Extinction debt is a one-way street, so we can't create new species

% by creating a negative extinction debt (not for a few million years, anyway)

if SppLost < 0, SppLost = 0; end

A(i+1) = A(i) - Degraded - Protected;

R(i+1) = R(i) + Protected + Recovered;

L(i+1) = L(i) + Degraded - Revegetated;

V(i+1) = V(i) + Revegetated - Recovered;

S(i+1) = S(i) - SppLost;

end

function Plotting_Routine(u,Cp,Cv,B,S_0,Alpha,z,theta,A_0,R_0,V_0,L_0,Spp,d,g,dt,T,LA,LV,LR,LS,ID)

% Calculate the total benefit using various allocation strategies,

% including the optimal strategy, protection only, restoration only, and

% zero allocation of funds (no action)

[A_Opt,R_Opt,V_Opt,L_Opt,Spp_Opt] = SubForce_AtlanticF(u,Alpha,S_0,z,theta,Cp,Cv,B,A_0,R_0,V_0,L_0,d,g,T,dt);

[A_Protect,R_Protect,V_Protect,L_Protect,Spp_Protect] = SubForce_AtlanticF(ones(size(u)),Alpha,S_0,z,theta,Cp,Cv,B,A_0,R_0,V_0,L_0,d,g,T,dt);

[A_Restore,R_Restore,V_Restore,L_Restore,Spp_Restore] = SubForce_AtlanticF(zeros(size(u)),Alpha,S_0,z,theta,Cp,Cv,B,A_0,R_0,V_0,L_0,d,g,T,dt);

[~,~,~,~,S_NoAction] = SubForce_AtlanticF(ones(size(u)),Alpha,S_0,z,theta,Cp,Cv,0,A_0,R_0,V_0,L_0,d,g,T,dt);

% Define a set of colours for the different stacked bar graphs

C(1,:) = 90.*[1 1 1]; C(1,:) = C(1,:)./255; % Available

C(2,:) = [0, 103, 1]; C(2,:) = C(2,:)./255; % Protected

C(3,:) = [28, 57, 187]; C(3,:) = C(3,:)./255; % Restoring

C(4,:) = [139, 0, 0 ]; C(4,:) = C(4,:)./255; % Degraded

C = C([2,3,1,4],:);

% Plotting routines

LabelySizey = 12; NumySizey = 9; ColorySizey = 11; LineyWidey = 2;

figure(3), clf

subplot('position',[0.1 0.28 0.33 0.62]), hold on, box on

TV = dt:dt:T; OV = cumsum([R_Opt; V_Opt; A_Opt; L_Opt]);

for i = 4:-1:1; B1 = bar(TV,100.*OV(i,:),1); set(B1,'edgecolor','none','facecolor',C(i,:)); end

xlim([0 T]), ylim([0 100]), xlabel('Time (years)','fontsize',LabelySizey)

ylabel('Habitat state (%)','fontsize',LabelySizey)

set(gca,'fontsize',NumySizey,'ytick',[0:25:100])

text(-8.5,100,'A','fontsize',LabelySizey)

LW = 8;

xx = [0.08 0.35 0.46]; subplot('position',[0.1 0.92 0.33 0.05]), hold on, box off, axis off, set(gca,'xtick',[],'ytick',[])

text(0.1,0.5,'Protect','fontsize',LabelySizey-1,'fontweight','bold','color',C(1,:))

plot([0 xx(1)],[0.5 0.5],'color',C(1,:),'linewidth',LW)

plot([xx(2) xx(3)],[0.5 0.5],'color',C(1,:),'linewidth',LW)

plot([0 0],[0.25 0.75],'color',C(1,:),'linewidth',LW)

plot([xx(3) xx(3)],[0.25 0.75],'color',C(1,:),'linewidth',LW)

xlim([-0.01 1.01]), ylim([0 1])

xx = [0.48 0.59 0.89];

text(0.61,0.5,'Restore','fontsize',LabelySizey-1,'fontweight','bold','color',C(2,:))

plot([xx(1) xx(2)],[0.5 0.5],'color',C(2,:),'linewidth',LW)

plot([xx(3) 1],[0.5 0.5],'color',C(2,:),'linewidth',LW)

plot([xx(1) xx(1)],[0.25 0.75],'color',C(2,:),'linewidth',LW)

plot([1 1],[0.25 0.75],'color',C(2,:),'linewidth',LW)

xlim([-0.01 1.01]), ylim([0 1])

subplot('position',[0.56 0.28 0.33 0.62]), hold on, box on

TV = dt:dt:T; OV = cumsum([R_Protect; V_Protect; A_Protect; L_Protect]);

for i = 4:-1:1; B1 = bar(TV,100.*OV(i,:),1); set(B1,'edgecolor','none','facecolor',C(i,:)); end

xlim([0 T]), ylim([0 100]), xlabel('Time (years)','fontsize',LabelySizey)

ylabel('Habitat state (%)','fontsize',LabelySizey)

set(gca,'fontsize',NumySizey,'ytick',[0:25:100])

text(-8.5,100,'B','fontsize',LabelySizey)

xx = [0.35 0.65]; LW = 8;

subplot('position',[0.56 0.92 0.33 0.05]), hold on, box off, axis off, set(gca,'xtick',[],'ytick',[])

text(0.39,0.5,'Protect','fontsize',LabelySizey-1,'fontweight','bold','color',C(1,:))

plot([0 xx(1)],[0.5 0.5],'color',C(1,:),'linewidth',LW)

plot([xx(2) 1],[0.5 0.5],'color',C(1,:),'linewidth',LW)

plot([0 0],[0.25 0.75],'color',C(1,:),'linewidth',LW)

plot([1 1],[0.25 0.75],'color',C(1,:),'linewidth',LW)

xlim([-0.01 1.01]), ylim([0 1])

% % % Uncomment the following lines to output figures as a CSV

% headers = {'Time';'OPTIMAL Protected habitat (proportion)';...

% 'OPTIMAL Restoring habitat (proportion)';...

% 'OPTIMAL Unprotected habitat (proportion)';...

% 'OPTIMAL Degraded habitat (proportion)';...

% 'PROTECT Protected habitat (proportion)';...

% 'PROTECT Restoring habitat (proportion)';...

% 'PROTECT Unprotected habitat (proportion)';...

% 'PROTECT Degraded habitat (proportion)';...

% 'RESTORE Protected habitat (proportion)';...

% 'RESTORE Restoring habitat (proportion)';...

% 'RESTORE Unprotected habitat (proportion)';...

% 'RESTORE Degraded habitat (proportion)'};

% Output_data = [[dt:dt:T]; [R_Opt; V_Opt; A_Opt; L_Opt; R_Protect; V_Protect; A_Protect; L_Protect; R_Restore; V_Restore; A_Restore; L_Restore]]';

% csvwrite_with_headers('Figure_3_data.csv',Output_data,headers)

figure(2)

subplot('position',[0.15 0.07 0.8 0.4]), cla, hold on

Relative_Performance_of_Restoration = 100*(1-(Alpha-Spp_Restore)./(Alpha-S_NoAction));

Relative_Performance_of_Protection = 100*(1-(Alpha-Spp_Protect)./(Alpha-S_NoAction));

Relative_Performance_of_Optimal = 100*(1-(Alpha-Spp_Opt)./(Alpha-S_NoAction));

plot(dt:dt:T,Relative_Performance_of_Restoration,'-','linewidth',LineyWidey+3,'color',C(2,:))

plot(dt:dt:T,Relative_Performance_of_Protection,'-','linewidth',LineyWidey+2,'color',C(1,:))

% Have to muck around with the plotting of dashed lines in high-res TIFF files outputted by matlab

YY = 100*(1-(Alpha-Spp_Opt)./(Alpha-S_NoAction)); SS = 15;

YY(1:SS:end) = nan; YY(2:SS:end) = nan; YY(3:SS:end) = nan; YY(4:SS:end) = nan;

plot(dt:dt:T,YY, '-','linewidth',LineyWidey+1,'color','k')

xlabel('Time (years)','fontsize',LabelySizey), xlim([0 T])

ylabel('Averted species loss (%)','fontsize',LabelySizey),

ylim([0 20]); L = legend('Restoration','Protection','Opt. switch',2); set(L,'fontsize',12)

text(-3,20,'B','fontsize',LabelySizey); set(gca,'fontsize',NumySizey,'ytick',[0:5:100]), box on

% % Uncomment the following lines to output figures as a CSV

% headers = {'Time';'Performance of restoration relative to no action';'Performance of protection relative to no action';'Performance of optimal solution relative to no action'};

% Output_data = [[dt:dt:T]; Relative_Performance_of_Restoration; Relative_Performance_of_Protection; Relative_Performance_of_Optimal]';

% csvwrite_with_headers('Figure_2B_data.csv',Output_data,headers)

% % Uncomment the following lines to output figures as TIFs

% set(gcf, 'paperunits', 'centimeters')

% set(gcf, 'PaperPositionMode', 'manual');

% set(gcf, 'paperposition', [0 0 17 17])

% print -dtiff -r300 Outcomes.tif

% Plot the stacked bars of the landscape dynamics

figure(3); XL = 0.1; YL = 0.06; SC = 1.2;

subplot('position',[XL YL 0.05 0.05]), box off

P1 = patch([0 0 1 1],[0 1 1 0],C(1,:)), set(gca,'xtick',[],'ytick',[])

text(1.2,0.5,'Protected','fontsize',ColorySizey)

XL = XL+0.168*SC;

subplot('position',[XL YL 0.05 0.05]), box off

P1 = patch([0 0 1 1],[0 1 1 0],C(2,:)), set(gca,'xtick',[],'ytick',[])

text(1.2,0.5,'Restoring','fontsize',ColorySizey)

XL = XL+0.168*SC;

subplot('position',[XL YL 0.05 0.05]), box off

P1 = patch([0 0 1 1],[0 1 1 0],C(3,:)), set(gca,'xtick',[],'ytick',[])

text(1.2,0.5,'Unprotected','fontsize',ColorySizey)

XL = XL+0.19*SC;

subplot('position',[XL YL 0.05 0.05]), box off

P1 = patch([0 0 1 1],[0 1 1 0],C(4,:)), set(gca,'xtick',[],'ytick',[])

text(1.2,0.5,'Degraded','fontsize',ColorySizey)

XL = XL+0.17*SC;

set(gcf, 'paperunits', 'centimeters')

set(gcf, 'PaperPositionMode', 'manual');

set(gcf, 'paperposition', [0 0 17 9])

print -dtiff -r400 ../Figures/Figure_2.tif
